# Pyrrolidine-Mediated Direct Preparation of (*E*)-Monoarylidene Derivatives of Homo- and Heterocyclic Ketones with Various Aldehydes

**DOI:** 10.3390/molecules19021976

**Published:** 2014-02-12

**Authors:** Xin Gu, Xiaoyan Wang, Fengtian Wang, Hongbao Sun, Jie Liu, Yongmei Xie, Mingli Xiang

**Affiliations:** 1 State Key Laboratory of Biotherapy, West China Hospital, West China Medical School, Sichuan University, Chengdu 610041, China; E-Mails: xingu2014@yeah.net (X.G.); ftwang2014@163.com (F.W.); hongbaosun88@gmail.com (H.S.); mlxiang123@163.com (M.X.); 2 Analytical & Testing Center, Sichuan University, Chengdu 610064, China; E-Mail: wangxiaoyan@scu.edu.cn

**Keywords:** α,β-unsaturated ketones, pyrrolidine, 1-methyl-4-piperidone, Mannich-elimination sequence, Claisen-Schmidt condensation

## Abstract

An efficient method for the facile synthesis of (*E*)-monoarylidene derivatives of homo- and heterocyclic ketones with various aldehydes in the presence of a pyrrolidine organocatalyst has been achieved. A range of α,β-unsaturated ketones were obtained in moderate to high yields (up to 99%). Unlike the Claisen-Schmidt condensation process, the formation of undesired bisarylidene byproducts is not observed. The possible reaction mechanism suggests that the reaction proceeds via a Mannich-elimination sequence.

## 1. Introduction

**Scheme 1:**
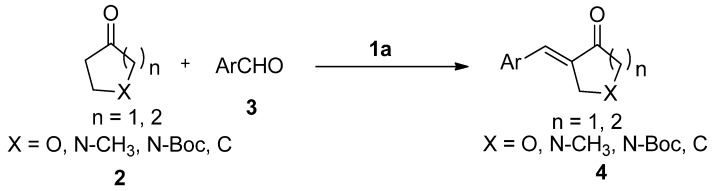
Reaction of ketone **2** with aldehydes **3**.

α,β-Unsaturated ketones represent an important class of compounds, as they possess a broad spectrum of biological activity such as anticancer, cytotoxic, anti-inflammatory, analgesic, and antipyretic behavior [1,2,3]. In addition, these compounds are also useful synthons for the preparation of different functionalized organic compounds, and have been widely used for this purpose in organic synthesis [4,5,6]. Thus, the synthesis of these compounds has attracted increasing attention from chemists, biochemists and pharmacologists. So far, several strategies are reported for the preparation of these compounds, which are accomplished by various methods such as condensation, oxidation, elimination, acylation, and insertion of carbon monoxide, among others [7,8,9,10,11,12,13,14,15]. In connection with a project in our laboratory, we required mono-2-arylidene derivatives of ketones, particularly of piperidone. The ideal choice in our case is to form the mono-2-arylidene structural unit via a Claisen-Schmidt condensation between the parent ketones and aryl aldehydes [16,17,18,19]. However, this method employs a relatively strong base such as a metal hydroxide or metal alkoxide, so it is often accompanied by side reactions and it offers narrow substrate diversity. Several publications [20,21,22,23,24] have demonstrated that such reactions proceed beyond mono-condensation and in many cases bisarylmethylidenes of various homo- and heterocyclic ketones can be formed exclusively, even when the molar ratio of starting aldehyde to ketone is substantially below 1:1. Accordingly, the development of an efficient catalyst for the direct preparation of the monoarylmethylidenes of various homo- and heterocyclic ketones is a challenge and has become a much attempted sought after endeavor. As an alternative, mild Lewis acid-catalyzed tandem Mukaiyama aldol-dehydration reactions have been described [25,26,27], but silylation of the ketone introduces another step and reduces the atom economy [28]. To overcome this limitation, recent improved approaches employing different catalytic system have been reported [29,30,31,32]. These methods are applicable to the synthesis of mono-2-arylidene derivatives of cyclohexanone, cyclopentanone, tetrahydrothiapyrone and aliphatic ketones, but only a few references for the preparation of mono-2-arylidene derivatives of piperidone were found. In these systems, MgBr_2_·OEt_2_/TMSNMe_2_, microwave irradiation and ultrasound were employed [31,32,33]. Piperidone is an important structural motif, often found in bioactive molecules. The use of such a ketone for the production of new α,β-unsaturated ketones of biological interest is not very extensive. Recently, chiral secondary amines were used as organocatalysts in the direct aldol reaction with great success [34,35,36,37]. The mechanism suggesting that ketones and amines facilicate the formation of the intermediate enamine, encouraged us to study the direct aldol-dehydration reaction for producing the title compounds. As a part of our own interest in aldol dehydration reactions, we report herein an efficient procedure for the synthesis of (*E*)-mono-2-arylidene derivatives of piperidone. The procedure is also applicable to the reactions of other homo- and heterocyclic ketones with various aldehydes (Scheme 1).

## 2. Results and Discussion

**Figure 1:**
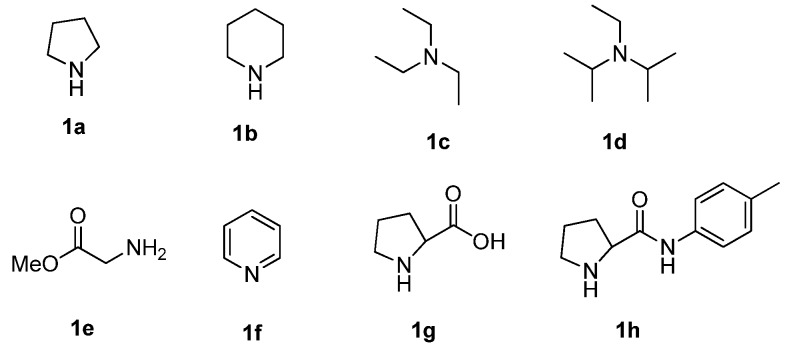
Structures of the catalysts studied.

Initially, the reaction between 1-methyl-4-piperidone (**2a**) and benzaldehyde (**3a**) was selected as a benchmark for catalyst evaluation (Figure 1). Some screening results are listed in Table 1. Initial studies showed that 20 mol% of pyrrolidine (**1a**) could catalyze the reaction to afford (*E*)-3-arylidene-1-methyl-4-piperidone (**4a**) as a single product in 46% yield (Table 1, entry 1), but a large amount of starting materials remained unreacted. Better results were obtained when the catalyst loading was increased to 1.2 equivalents, leading to the formation of **4a** in 77% yield (Table 1, entry 2). When other catalysts such as piperidine, Et_3_N, DIPEA, methyl glycinate, pyridine, proline and proline derivatives were screened, no better result was obtained (Table 1, entries 3-9). Methyl glycinate (**1e**), a primary amine, was employed as a catalyst too, and the monocondensation product **4a** was obtained in 22% yield (Table 1, entry 5). 

**Table 1:** Optimization of reaction conditions to yield compound **4a**^a^.

Entry	Catalyst	Solvent	Temp (°C)	Yield (%) ^b^
1	**1a**	CH_2_Cl_2_	25	46 ^c^
2	**1a**	CH_2_Cl_2_	25	77
3	**1b**	CH_2_Cl_2_	25	18 ^d^
4	**1c**	CH_2_Cl_2_	25	N.R ^e^
5	**1d**	CH_2_Cl_2_	25	N.R
6	**1e**	CH_2_Cl_2_	25	22
7	**1f**	CH_2_Cl_2_	25	N.R
8	**1g**	CH_2_Cl_2_	25	N.D ^f^
9	**1h**	CH_2_Cl_2_	25	trace
10	**1a**	CHCl_3_	25	42
11	**1a**	Et_2_O	25	41
12	**1a**	toluene	25	18
13	**1a**	dioxane	25	trace
14	**1a**	CH_3_OH	25	68
15	**1a**	EtOH	25	73
16	**1a**	EtOH/H_2_O	25	38 ^f^
17	**1a**	CH_2_Cl_2_	0	59
18	**1a**	CH_2_Cl_2_	40	94

^a^ Unless indicated otherwise, the reaction was carried out in 0.1 mmol scale in solvent (1.0 mL) for 4 h and the ratio of **1/2a/3a** is 1.2/1/1; ^b^ Isolated yield based on 1-methyl-4-piperidone; ^c^ Catalyst loading is 20 mol%; ^d^ Reaction time is 48 h; ^e^ N.R refers to no reaction; ^f^ N.D refers to not detected; ^g^ V_EtOH_ :V_H2O_ = 3:2.

Proline, a prominent catalyst, had been used previously in aldol reactions between cyclic ketones and aldehydes, but when proline was used in this reaction, no product was detected, and not even the aldol product was detected (Table 1, entry 8). All these results indicated that only the secondary amine **1a** was a suitable catalyst and it was selected as reaction catalyst for the subsequent investigations.

To further improve the yield, efforts were made to optimize other reaction parameters including solvents and reaction temperatures. Thus, the reaction was studied in different solvents that included CH_2_Cl_2_, CHCl_3_, Et_2_O, toluene, dioxane, CH_3_OH, EtOH and EtOH/H_2_O (Table 1, entries 10–16). Although the reaction solvents influenced the rate of the reaction, they did not affect the formation of (*E*)**-4a** as single product during the course of reaction, regardless of the protic or aprotic nature of the solvent. Aldol products and bisarylmethylidenes of piperidone were not observed. The temperature also influenced the rate of the reaction. Elevating the reaction temperature resulted in a high reactivity (Table 1, entries 17, 18), while conducting the reaction at 40 °C provided the best results. Through extensive screening, the optimized reaction conditions were found to be **2a**/**3a**/**1a** = 1/1/1.2 and 1.0 mL of CH_2_Cl_2_ as solvent at 40 °C.

The application scope of the catalytic system was then examined under the optimal conditions. As shown in Table 2, a variety of aromatic aldehydes bearing various substituents were investigated, and the corresponding products were obtained in moderate to high yields (up to 99%, Table 2, entries 1–18). The electronic properties and steric hindrance of the substituents at the aromatic ring affected the yields strongly (Table 2, entries 1–13). Aromatic aldehydes with electron-withdrawing groups gave higher yields than those with electron-donating groups (Table 2, entries 2–6, 10, 12, 13 *vs.* 7, 8, 11). *ortho*-Substituted aromatic aldehydes gave higher yields than *para*- and *meta*-substituted aromatic aldehydes (Table 2, entries entries 4, 5, 8 *vs.* 10–13). Naphthyl and heterocyclic aromatic aldehydes also participated in this reaction in moderate yields (Table 2, entries 14–18). Moreover, an aliphatic aldehyde was investigated and it was transformed with a yield of 51% (Table 2, entry 19).

To further extend the application of our procedure, the reactions of other ketones, such as cyclohexanone, cyclopentanone and 4-oxotetrahydropyran with several representative aldehydes were also examined (Table 2, entries 20–24). Interestingly, ketones with different structures worked well under the optimized conditions and these reactions gave the corresponding products in good yields. Similarly, the electronic nature of the substrate influenced the reactivity. Aromatic aldehydes with electron-withdrawing groups gave higher yields (Table 2, entry 21 *vs.* 22).

**Scheme 2:**
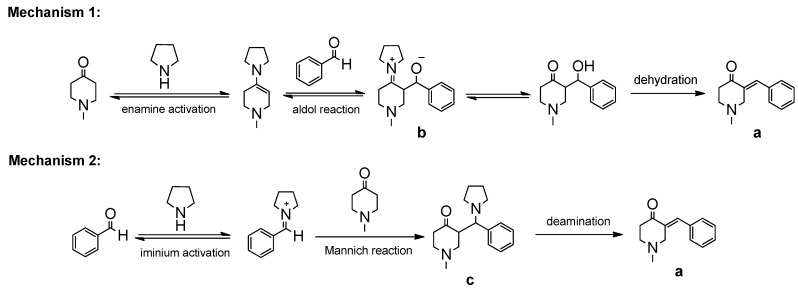
Two proposed mechanisms for the formation of α, β-unsaturated ketones.

Based on the results and previous reports [29,34,35,36,37,38], two possible reaction mechanisms for the formation of (*E*)-monoarylidene derivatives of homo- and heterocyclic ketones with various aldehydes have been proposed. As depicted in Scheme 2, one route is the generation through an aldol reaction of product **a**, which then undergoes a dehydration process (Scheme 2, mechanism 1). The reaction proceeded through a course of enamine activation. Another route is the generation of product **a** through a Mannich-elimination sequence (Scheme 2, mechanism 2). The 1-methyl-4-piperidone attacks the iminium complex formed from pyrrolidine and benzaldehyde to give intermidate **c**, which then undergoes a elimination process to afford product **a**. The iminium species formation is an important mode of activation and facilitates this reaction.

**Table 2:** Synthesis of monoarylmethylidenes of various homo- and heterocyclic ketones.

Entry	2	3	4	Yield (%) ^b^
1	X = N-CH_3_, *n* = 2	R_1_ = Ph	4a	94
2	X = N-CH_3_, *n* = 2	R_1_ = 4-NO_2_-Ph	4b	90
3	X = N-CH_3_, *n* = 2	R_1_ = 4-CN-Ph	4c	99
4	X = N-CH_3_, *n* = 2	R_1_ = 4-F-Ph	4d	75
5	X = N-CH_3_, *n* = 2	R_1_ = 4-Br-Ph	4e	73
6	X = N-CH_3_, *n* = 2	R_1_ = 3,4-diCl-Ph	4f	80
7	X = N-CH_3_, *n* = 2	R_1_ = 4-CH_3_-Ph	4g	54
8	X = N-CH_3_, *n* = 2	R_1_ = 4-CH_3_O-Ph	4h	50
9	X = N-CH_3_, *n* = 2	R_1_ = 3-Cl-Ph	4i	81
10	X = N-CH_3_, *n* = 2	R_1_ = 3-Br-Ph	4j	86
11	X = N-CH_3_, *n* = 2	R_1_ = 3-CH_3_O-Ph	4k	65
12	X = N-CH3, *n* = 2	R_1_ = 2-F-Ph	4l	84
13	X = N-CH_3_, *n* = 2	R_1_ = 2-Br-Ph	4m	94
14	X = N-CH_3_, *n* = 2	R_1_ = 2-naphthyl	4n	60
15	X = N-CH_3_,*n* = 2	R_1_ = 1-naphthyl	4o	93
16	X = N-CH_3_, *n* = 2	R_1_ = 2-pyridinyl	4p	78
17	X = N-CH_3_, *n* = 2	R_1_ = 4-pyridinyl	4q	59
18	X = N-CH_3_, *n* = 2	R_1_ = 2-thienyl	4r	46
19	X = N-CH_3_, *n* = 2	R_1_ = CH_3_CH_2_CH_2_	4s	53
20	X = N-Boc, *n* = 2	R_1_ = Ph	4t	92
21	X = O, *n* = 2	R_1_ = Ph	4u	53
22	X = O, *n* = 2	R_1_ = 4-NO_2_-Ph	4v	64
23	X = C, *n* = 2	R_1_ = 4-NO_2_-Ph	4w	84^c^
24	X = C, *n* = 1	R_1_ = 4-NO_2_-Ph	4x	95

^a^ Unless indicated otherwise, the reaction was carried out in 0.1 mmol scale in CH_2_Cl_2_ (1.0 mL) at 40 °C for 4 h, and the ratio of **1a**/**2/3** is 1.2/1/1; ^b^ Isolated yield based on ketones; ^c^ Reaction time is 20 h.

**Figure 2:**
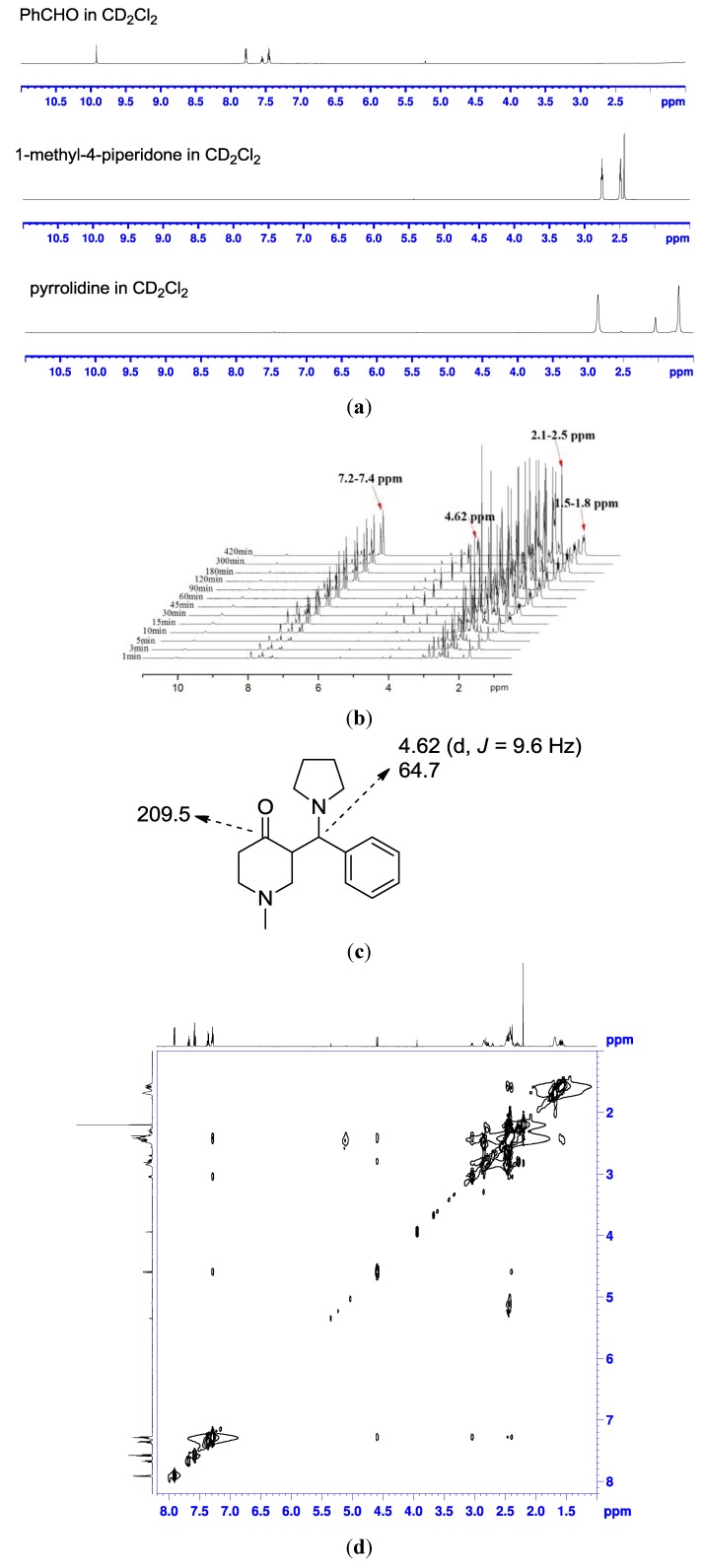
(**a**) ^1^H spectra of the starting material; (**b**) ^1^H spectra recorded at different time; (**c**) selected ^1^H and ^13^C chemical shift of **c**; and (**d**) The NOESY spectra recorded after 5 h.

To obtain a better view of the nature of the catalytic species at work in this reaction, first careful monitoring of the course of the reaction of 1-methyl-4-piperidone with benzaldehyde in the presence of 1.2 eq pyrrolidine in CH_2_Cl_2_ has been performed by TLC. During the course of the reaction, the aldol product was never detected. Therefore, we hypothesized the generation of product **a** may go through a Mannich-elimination sequence (Scheme 2, mechanism 2). We next validated the possible reaction mechanism for the formation of product **a** using some spectroscopic studies. NMR spectra of three raw materials are presented in Figure 2a. The ^1^H-NMR spectra were then recorded over time (Figure 2b). After 15 min, the appearance of multiple peaks in the δ 7.2–7.4, 2.1–2.5, 1.5–1.8 ppm region and a doublet at δ 4.62 ppm evidenced formation of the intermediate. From HSQC spectra, the carbon at 64.7 ppm was a typical signal which linked with the hydrogen at 4.62 ppm. As the reaction proceeded, the peaks of the signal became more apparent. After 5 h, the amount of intermediate did not increase. It is hard to distinguish whether the intermediate was **b** or **c** from the ^1^H-NMR spectra, but the ^13^C spectra provided some additional information. Three peaks at δ 209.5, 208.4 and 192.2 ppm were assigned to the carbonyl carbons of the two unreacted raw materials and the intermediate. A chemical shift in the 180-220 ppm range for the iminium carbon of intermediate **b** is not reasonable, therefore the intermediate should be **c**. The quantitative ^13^C-NMR results showed that the integration of the carbon at 209.5 ppm and the integration of the one at 64.7 ppm were approximately equal, suggesting the two carbons were those of intermediate **c** (Figure 2c). Moreover, the structure of intermediate **c** was also verified by A NOE experiment (Figure 2d). The obvious cross-peak between the pyrrole and the phenyl ring illustrated that the intermediate should be **c** instead of **b**. This, based on ^1^H-, ^13^C-, quantitative ^13^C-NMR, and NOESY data of the mixture, the structure of the intermediate was identified as **c** (Figure S3). However, we do not see evidence for product formation under these reaction conditions. It should be noted that although no products are evident in the solution during the NMR reactions, the intermediate turns into product during subsequent purification on silicagel.

## 3. Experimental

### 3.1. General

All chemicals were obtained from commercial sources and used without further purification. Column chromatography was carried out on silica gel (300–400 mesh, Qingdao Marine Chemical Ltd., Qingdao, China). Thin layer chromatography (TLC) was performed on TLC silica gel 60 F254 plates. ^1^H-NMR spectra were recorded on Bruker AVII-400 or 600 MHz instruments. The chemical shifts were recorded in ppm relative to tetramethylsilane and with the solvent (CDCl_3_) resonance as the internal standard. Data were reported as follows: chemical shift, multiplicity (s = singlet, d = doublet, t = triplet, q = quartlet, m = multiplet), coupling constants (Hz), integration. ^13^C-NMR data were collected at 100 or 150 MHz with complete proton decoupling. Chemical shifts were reported in ppm from tetramethylsilane with the solvent (CDCl_3_) resonance as internal standard. MS spectra were obtained on a Waters Quattro Premier XETM triple quadrupole mass spectrometer and methanol was used to dissolve the sample. Melting points were recorded on a SGW X-4 melting point instrument (Shanghai Precision & Scientific Instrument Co., Ltd, Shanghai, China).

### 3.2. General Experimental Procedure

A mixture of 1-methyl-4-piperidone (**2a**, 0.1 mmol) and pyrrolidine (0.24 mmol) in CH_2_Cl_2_ (1.0 mL) was stirred about 5 min at room temperature. Then, benzaldehyde (**3a**, 0.1 mmol) was added and the mixture was stirred for 4 h at 40 °C. After completion of the reaction (TLC), the solvent was removed under vacuum. The crude product was subjected to column chromatography on silica gel using petroleum ether/ethyl acetate/triethylamine (PE/EA/TEA = 3:1:0.04) as the eluent to give **4a**. The compounds **4b**–**x** were synthesized by a similar procedure as described for compound **4a**.

### 3.3. Spectral Data

*(E)-3-Benzylidene-1-methylpiperidin-4-one* (**4a**). Yield 94%; Brown liquid; ^1^H-NMR (600 MHz): δ 2.44 (s, 3H), 2.67 (t, *J* = 6.0 Hz, 2H), 2.81 (d, *J* = 6.0 Hz, 2H), 3.65 (s, 2H), 7.34–7.41 (m, 5H), 7.58 (s, 1H); ^13^C-NMR (150 MHz): δ 39.1, 46.2, 52.8, 57.7, 128.5, 129.1, 130.4, 133.0, 134.9, 135.9, 197.8; MS: *m/z* 202 [M+H]^+^.

*(E)-1-Methyl-3-(4-nitrobenzylidene)piperidin-4-one* (**4b**). Yield 90%; Yellow solid; m.p. 141–142 °C; ^1^H-NMR (400 MHz): δ 2.45 (s, 3H), 2.71 (t, *J =* 6.0 Hz, 2H), 2.85 (t, *J =* 6.0 Hz, 2H), 3.62 (s, 2H), 7.48 (d, *J =* 8.4 Hz, 2H), 7.55 (s, 1H), 8.26 (d, *J =* 8.4 Hz, 2H); ^13^C-NMR (100 MHz): δ 39.2, 46.2, 52.7, 57.5, 123.7, 130.8, 132.8, 136.0, 141.3, 147.5, 197.3; MS: *m/z* 247 [M+H]^+^.

*(E)-4-((1-Methyl-4-oxopiperidin-3-ylidene)methyl)benzonitrile* (**4c**). Yield 99%; Yellow solid; m.p. 122–123 °C; ^1^H-NMR (400 MHz): δ 2.45 (s, 3H), 2.70 (t, *J =* 6.0 Hz, 2H), 2.84 (t, *J =* 6.0 Hz, 2H), 3.60 (s, 2H), 7.42 (d, *J =* 8.0 Hz, 2H), 7.51 (s, 1H), 7.69 (d, *J =* 8.0 Hz, 2H); ^13^C-NMR (100 MHz): δ 39.1, 46.1, 52.6, 57.4, 112.3, 118.4, 130.5, 132.2, 133.2, 135.6, 139.4, 197.3; MS: *m/z* 227 [M+H]^+^.

*(E)-3-(4-Fluorobenzylidene)-1-methylpiperidin-4-one* (**4d**). Yield 75%; Yellow solid; m.p. 38–39 °C; ^1^H-NMR (400 MHz): δ 2.45 (s, 3H), 2.67 (t, *J =* 6.0 Hz, 2H), 2.82 (t, *J =* 6.0 Hz, 2H), 3.62 (s, 1H), 3.63 (s, 1H), 7.08–7.12 (m, 2H), 7.32–7.35 (m, 2H), 7.53 (s, 1H); ^13^C-NMR (100 MHz): δ 39.1, 46.2, 52.7, 57.6, 115.7 (d, *J =* 22 Hz), 131.0 (d, *J =* 3 Hz), 132.3 (d, *J =* 8 Hz), 132.7 (d, *J =* 1 Hz), 134.7, 162.9 (d, *J =* 250 Hz), 197.6; MS: *m/z* 220 [M+H]^+^.

*(E)-3-(4-Bromobenzylidene)-1-methylpiperidin-4-one* (**4e**). Yield 73%; Yellow solid; m.p. 63–64 °C; ^1^H-NMR (400 MHz): δ 2.44 (s, 3H), 2.67 (t, *J =* 6.0 Hz, 2H), 2.82 (t, *J =* 6.0 Hz, 2H), 3.59 (s, 1H), 3.60 (s, 1H), 7.20 (d, *J =* 8.4 Hz, 2H), 7.48 (s, 1H), 7.53 (d, *J =* 8.4 Hz, 2H); ^13^C-NMR (100 MHz): δ 39.1, 46.2, 52.8, 57.7, 123.4, 131.8, 131.8, 133.6, 133.8, 134.5, 197.6; MS: *m/z* 302 [M+Na]^+^.

*(E)-3-(3,4-Dichlorobenzylidene)-1-methylpiperidin-4-one* (**4f**). Yield 80%; Yellow solid; m.p. 73–74 °C; ^1^H-NMR (400 MHz): δ 2.45 (s, 3H), 2.68 (t, *J =* 6.0 Hz, 2H), 2.82 (t, *J =* 6.0 Hz, 2H), 3.59 (s, 1H), 3.60 (s, 1H), 7.15–7.18 (m, 1H), 7.41–7.43 (m, 2H), 7.47–7.49 (m, 1H); ^13^C-NMR (100 MHz): δ 39.2, 46.2, 52.7, 57.5, 129.4, 130.6, 131.8, 132.9, 133.1, 133.2, 134.5, 134.9, 197.4; MS: *m/z* 270 [M]^+^.

*(E)-1-Methyl-3-(4-methylbenzylidene)piperidin-4-one* (**4g**). Yield 54%; Brown liquid; ^1^H-NMR (400 MHz): δ 2.37 (s, 3H), 2.44 (s, 3H), 2.66 (t, *J =* 6.0 Hz, 2H), 2.80 (t, *J =* 6.0 Hz, 2H), 3.65 (s, 1H), 3.66 (s, 1H), 7.20 (d, *J =* 8.0 Hz, 2H), 7.25 (d, *J =* 8.4 Hz, 2H), 7.56 (s, 1H); ^13^C-NMR (100 MHz): δ 21.3, 39.0, 46.2, 52.7, 57.8, 129.2, 130.5, 132.0, 132.1, 136.0, 139.3, 197.6; MS: *m/z* 216 [M+H]^+^.

*(E)-3-(4-Methoxybenzylidene)-1-methylpiperidin-4-one* (**4h**). Yield 50%; Yellow solid; m.p. 60–61 °C. ^1^H-NMR (400 MHz): δ 2.46 (s, 3H), 2.66 (t, *J =* 6.0 Hz, 2H), 2.81 (t, *J =* 6.0 Hz, 2H), 3.66 (s, 2H), 3.84 (s, 3H), 6.93 (d, *J =* 8.8 Hz, 2H), 7.33 (d, *J =* 8.4Hz, 2H), 7.56 (s, 1H); ^13^C-NMR (100 MHz): δ 39.1, 46.3, 52.7, 55.4, 58.0, 114.1, 127.6, 130.9, 132.3, 132.5, 136.0, 160.4, 197.7. MS: *m/z* 232 [M+H]^+^.

*(E)-3-(3-Chlorobenzylidene)-1-methylpiperidin-4-one* (**4i**). Yield 81%; Yellow solid; m.p. 57–58 °C; ^1^H-NMR (400 MHz): δ 2.45 (s, 3H), 2.68 (t, *J =* 6.0 Hz, 2H), 2.82 (t, *J =* 6.0 Hz, 2H), 3.61 (s, 1H), 3.62 (s, 1H), 7.20–7.22 (m, 1H), 7.31–7.34 (m, 3H), 7.48 (s, 1H); ^13^C-NMR (100 MHz): δ 39.1, 46.2, 52.7, 57.5, 128.4, 129.0, 129.8, 129.9, 134.1, 134.2, 134.5, 136.7, 197.5; MS: *m/z* 235 [M+H]^+^.

*(E)-3-(3-Bromobenzylidene)-1-methylpiperidin-4-one* (**4j**). Yield 86%; Yellow solid; m.p. 51–52 °C; ^1^H-NMR (400 MHz): δ 2.44 (s, 3H), 2.67 (t, *J =* 6.0 Hz, 2H), 2.82 (t, *J =* 6.0 Hz, 2H), 3.60 (s, 1H), 3.61 (s, 1H), 7.26–7.27 (m, 2H), 7.46–7.48 (m, 3H); ^13^C-NMR (100 MHz): δ 39.1, 46.1, 52.7, 57.4, 122.5, 128.7, 130.0, 131.8, 132.8, 133.9, 134.2, 136.9, 197.4; MS: *m/z* 302 [M+Na]^+^.

*(E)-3-(3-Methoxybenzylidene)-1-methylpiperidin-4-one* (**4k**). Yield 65%; Brown liquid; ^1^H-NMR (400 MHz): δ 2.44 (s, 3H), 2.67 (t, *J =* 6.0 Hz, 2H), 2.81 (t, *J =* 6.0 Hz, 2H), 3.64 (s, 2H), 3.82 (s, 3H), 6.87–6.95 (m, 3H), 7.30–7.34 (m, 1H), 7.54 (s, 1H); ^13^C-NMR (100 MHz): δ 39.1, 46.2, 52.8, 55.3, 57.7, 114.6, 115.9, 122.8, 129.5, 133.2, 135.8, 136.2, 159.5, 197.8; MS: *m/z* 232 [M+H]^+^.

*(E)-3-(2-Fluorobenzylidene)-1-methylpiperidin-4-one* (**4l**). Yield 84%; Brown liquid; ^1^H-NMR (400 MHz): δ 2.42 (s, 3H), 2.68 (t, *J =* 6.0 Hz, 2H), 2.82 (t, *J =* 6.0 Hz, 2H), 3.52 (s, 2H), 7.08–7.18 (m, 2H), 7.22–7.28 (m, 1H), 7.32–7.37 (m, 1H), 7.61 (s, 1H);^ 13^C-NMR (100 MHz): δ 39.2, 46.1, 53.0, 57.5, 115.8 (d, *J =* 21 Hz), 122.8, 123.8, 128.3, 130.8 (d, *J =* 24 Hz), 130.9, 135.0, 160.9 (d, *J =* 250 Hz), 197.4; MS: *m/z* 220 [M+H]^+^.

*(E)-3-(2-Bromobenzylidene)-1-methylpiperidin-4-one* (**4m**). Yield 94%; Brown liquid; ^1^H-NMR (400 MHz): δ 2.39 (s, 3H), 2.69 (t, *J =* 6.0 Hz, 2H), 2.82 (t, *J =* 6.0 Hz, 2H), 3.46 (s, 2H), 7.16–7.22 (m, 2H), 7.28–7.34 (m, 1H), 7.60–7.63 (m, 2H); ^13^C-NMR (100 MHz): δ 39.3, 46.0, 53.1, 57.1, 126.8, 127.0, 130.2, 130.4, 133.1, 134.3, 134.8, 135.2, 197.6; MS: *m/z* 302 [M+Na]^+^.

*(E)-1-Methyl-3-(naphthalen-2-ylmethylene)piperidin-4-one* (**4n**). Yield 60%; Brown liquid; ^1^H-NMR (400 MHz): δ 2.45 (s, 3H), 2.70 (t, *J =* 6.0 Hz, 2H), 2.83 (t, *J =* 6.0 Hz, 2H), 3.74 (s, 1H), 3.75 (s, 1H), 7.43–7.45 (m, 1H), 7.50–7.53 (m, 2H), 7.73 (s, 1H), 7.80–7.87 (m, 4H); ^13^C-NMR (100 MHz): δ 39.1, 46.2, 52.8, 57.8, 126.6, 127.1, 127.4, 127.7, 128.2, 128.5, 130.5, 132.4, 133.0, 133.2, 133.3, 136.1, 197.7; MS: *m/z* 274 [M+Na]^+^.

*(E)-1-Methyl-3-(naphthalen-1-ylmethylene)piperidin-4-one* (**4o**). Yield 93%; Yellow solid; m.p. 62–63 °C; ^1^H-NMR (400 MHz): δ 2.35 (s, 3H), 2.74 (t, *J =* 6.0 Hz, 2H), 2.83 (t, *J =* 6.0 Hz, 2H), 3.49 (s, 2H), 7.29–7.30 (m, 1H), 7.45–7.53 (m, 3H), 7.85–7.88 (m, 2H), 7.94–7.96 (m, 1H), 8.13 (s, 1H); ^13^C-NMR (100 MHz): δ 39.4, 46.0, 53.3, 57.6, 124.7, 124.9, 126.3, 126.6, 126.8, 128.6, 129.4, 131.9, 132.0, 133.5, 134.0, 134.9, 197.8; MS: *m/z* 252 [M+H]^+^.

*(E)-1-Methyl-3-(pyridin-2-ylmethylene)piperidin-4-one* (**4p**). Yield 78%; Yellow solid; m.p. 120–121 °C; ^1^H-NMR (400 MHz): δ 2.48 (s, 3H), 2.70 (t, *J =* 6.0 Hz, 2H), 2.82 (t, *J =* 6.0 Hz, 2H), 4.07 (s, 1H), 4.08 (s, 1H), 7.18–7.21 (m, 1H), 7.40–7.44 (m, 2H), 7.67–7.72 (m, 1H), 8.69–8.70 (m, 1H); ^13^C-NMR (100 MHz): δ 39.3, 46.2, 52.5, 58.1, 122.7, 127.6, 132.3, 136.2, 136.5, 149.5, 154.6, 198.4; MS: *m/z* 203 [M+H]^+^.

*(E)-1-Methyl-3-(pyridin-4-ylmethylene)piperidin-4-one* (**4q**). Yield 59%; Brown liquid; ^1^H-NMR (400 MHz): δ 2.44 (s, 3H), 2.70 (t, *J =* 6.0 Hz, 2H), 2.84 (t, *J =* 6.0 Hz, 2H), 3.60 (s, 1H), 3.61 (s, 1H), 7.18–7.20 (m, 2H), 7.42–7.43 (m, 1H), 8.65–8.67 (m, 2H); ^13^C-NMR (100 MHz): δ 39.2, 46.1, 52.7, 57.3, 123.9, 132.3, 136.5, 142.3, 150.1, 197.3; MS: *m/z* 203 [M+H]^+^.

*(E)-1-Methyl-3-(thiophen-2-ylmethylene)piperidin-4-one* (**4r**). Yield 46%; Brown liquid; ^1^H-NMR (400 MHz): δ 2.52 (s, 3H), 2.66 (t, *J =* 6.0 Hz, 2H), 2.81 (t, *J =* 6.0 Hz, 2H), 3.68 (s, 1H), 3.69 (s, 1H), 7.13–7.16 (m, 1H), 7.32–7.33 (m, 1H), 7.56–7.57 (m, 1H), 7.78–7.80 (m, 1H); ^13^C-NMR (100 MHz): δ 39.0, 46.4, 52.3, 57.8, 128.0, 128.4, 129.5, 130.8, 133.5, 138.3, 196.9; MS: *m/z* 208 [M+H]^+^.

*(E)-3-Butylidene-1-methylpiperidin-4-one* (**4s**). Yield 53%; Brown liquid; ^1^H-NMR (400 MHz): δ 0.94 (t, *J =* 7.2 Hz, 3H), 1.47–1.52 (m, 2H), 2.06–2.07 (m, 2H), 2.44 (s, 3H), 2.56 (t, *J =* 6.0 Hz, 2H), 2.73 (t, *J =* 6.0 Hz, 2H), 3.33 (s, 2H), 6.68–6.73 (m, 1H); ^13^C-NMR (100 MHz): δ 13.9, 21.6, 29.7, 39.0, 46.2, 52.8, 56.0, 133.1, 140.0, 197.2; MS: *m/z* 168 [M+H]^+^.

*(E)-Tert-butyl 3-benzylidene-4-oxopiperidine-1-carboxylate* (**4t**). Yield 92%; Yellow solid; m.p. 107–108 °C. ^1^H-NMR (400 MHz): δ 1.44 (s, 9H), 2.66 (t, *J =* 6.0 Hz, 2H), 3.78 (t, *J =* 6.0 Hz, 2H), 4.69 (s, 2H), 7.37–7.42 (m, 5H), 7.63 (s, 1H);^ 13^C-NMR (100 MHz): 28.3, 39.1, 40.9, 44.9, 80.5, 128.7, 129.5, 130.5, 131.8, 134.4, 137.2, 154.5, 197.4; MS: *m/z* 310 [M+Na]^+^.

*(E)-3-Benzylidenedihydro-2H-pyran-4(3H)-one* (**4u**). Yield 53%; Yellow solid; m.p. 96–97 °C; ^1^H-NMR (600 MHz): δ 2.70 (t, *J =* 6.0 Hz, 2H), 4.09 (t, *J =* 6.0 Hz, 2H), 4.87 (s, 2H), 7.28–7.30 (m, 2H), 7.38–7.43 (m, 3H), 7.64 (s, 1H);^ 13^C-NMR (150 MHz): δ 39.8, 65.6, 68.7, 128.7, 129.5, 130.6, 133.3, 134.3, 136.2, 196.2; MS: *m/z* 211 [M+Na]^+^.

*(E)-3-(4-Nitrobenzylidene)dihydro-2H-pyran-4(3H)-one* (**4v**). Yield 64%; Yellow solid; m.p. 197–198 °C; ^1^H-NMR (400 MHz): δ 2.74 (t, *J =* 6.0 Hz, 2H), 4.11 (t, *J =* 6.0 Hz, 2H), 4.83 (s, 1H), 4.84 (s, 1H), 7.43 (d, *J =* 8.8 Hz, 2H), 7.62 (s, 1H), 8.27 (d, *J =* 8.8 Hz, 2H);^ 13^C-NMR (100 MHz): δ 39.9, 65.6, 68.4, 123.9, 130.9, 132.9, 136.4, 140.7, 147.8, 195.5; MS: *m/z* 256 [M+Na]^+^.

*(E)-2-(4-Nitrobenzylidene)cyclohexanone* (**4w**). Yield 84%; Yellow solid; m.p. 119–120 °C; ^1^H-NMR (400 MHz): δ 1.78–1.84 (m, 2H), 1.94–2.00 (m, 2H), 2.58 (t, *J =* 6.8 Hz, 2H), 2.82 (t, *J =* 6.8 Hz, 2H), 7.46 (s, 1H), 7.52 (d, *J =* 8.8 Hz, 2H), 8.24 (d, *J =* 8.8 Hz, 2H); ^13^C-NMR (100 MHz): δ 23.3, 23.8, 29.1, 40.5, 123.6, 130.7, 132.5, 140.0, 142.2, 147.3, 201.2; MS: *m/z* 232 [M+H]^+^. 

*(E)-2-(4-Nitrobenzylidene)cyclopentanone* (**4x**). Yield 95%; Yellow solid; m.p. 139–140 °C; ^1^H-NMR (400 MHz): δ 2.05–2.13 (m, 2H), 2.45–2.49 (m, 2H), 3.00–3.03 (m, 2H), 7.39–7.40 (m, 1H), 7.67 (d, *J =* 8.8 Hz, 2H), 8.27 (d, *J =* 8.8 Hz, 2H);^ 13^C-NMR (100 MHz): δ 20.1, 29.4, 37.7, 123.9, 129.3, 130.8, 139.9, 142.0, 147.6, 207.3; MS: *m/z* 240 [M+Na]^+^.

## 4. Conclusions

In conclusion, we have developed an efficient method for the direct preparation of (*E*)-mono-arylidene derivatives of homo- and heterocyclic ketones with various aldehydes. A range of α,β-unsaturated ketones were obtained in moderate to high yields (up to 99%). The reaction is simple and convenient, with mild reaction conditions using a catalyst that is readily available, which makes it useful. The possible reaction mechanism suggests that the reaction proceeds via a Mannich-elimination sequence. These monoarylidene derivatives are versatile intermediates by virtue of the range of possible subsequent transformations to other functional groups. Further study on the antibacterial and antitumor activities of these compounds is underway. 

## Supplementary Materials

Supplementary materials can be accessed at: http://www.mdpi.com/1420-3049/19/2/1976/s1.
