# Supplementary material for: Pyrrolidine-Mediated Direct Preparation of (E)-Monoarylidene Derivatives of Homo- and Heterocyclic Ketones with Various Aldehydes
Source: Molecules. 2014 Feb 12;19(2):1976–89. doi: 10.3390/molecules19021976 (PMC6271378; doi:10.3390/molecules19021976)
Supplement: Supplementary file 1 [file molecules-19-01976-s001.pdf]

Supplementary File

Contents

(A) General information……………………………………………………………1
(B) General procedure for the Synthesis of mono-arylidene derivatives ……......……2
(C) Spectral characterization data for mono-arylidene derivatives …………………...2
(D) Copies of NMR spectra for mono-arylidene derivatives…………………………6
(E) Configuration of the mono-arylidene derivatives…………………………………30
(F) Copies of NMR spectra for intermediate……………………………………….31

(A) General Information

Melting points were recorded at SGW X-4 Melting point instrument (Shanghai precision & scientific instrument Co., Ltd, Shanghai, China). ^{1}H-NMR spectra were recorded at Bruker AVII-400 or 600 MHz. The chemical shifts were recorded in ppm relative to tetramethylsilane and with the solvent resonance as the internal standard. Data were reported as follows: chemical shift, multiplicity (s = singlet, d = doublet, t = triplet, q = quartlet, m = multiplet), coupling constants (Hz), integration. ^{13}C-NMR data were collected at 100 or 150 MHz with complete proton decoupling. Chemical shifts were reported in ppm from the tetramethylsilane with the solvent resonance as internal standard. MS spectra were obtained on Waters Quattro Premier XETM triple quadrupole mass spectrometer and methanol was used to dissolve the sample. All chemicals were obtained from commercial sources and used without further purification. Column chromatography was carried out on silica gel (300–400 mesh, Qingdao Marine Chemical Ltd., Qingdao, China). Thin layer chromatography (TLC) was performed on TLC silica gel 60 F254 plates.

(B) General Procedure for the Synthesis of Mono-Arylidene Derivatives

Typical experimental procedure for **4a**:

A mixture of 1-methyl-4-piperidone **2a** (0.1 mmol) and pyrrolidine (0.24 mmol) in 1.0 mL CH_2Cl_2 was stirred about 5 minat room temperature. Then, benzaldehyde **3a** (0.1mmol) was added and the mixture was stirred for 4 h at 40 ℃. After completion of the reaction (TLC), the solvent was removed under vacuum. The crude product was subjected to column chromatography on silica gel using petroleum ether/ethyl acetate/triethylamine (PE/EA/TEA = 3:1:0.04) as the eluent to give **4a**.

Compounds **4b**–**x** were synthesized by a similar procedure as described for compound **4a**.

(C) Spectral Characterization Data for Mono-Arylidene Derivatives

(*E*)-*3-benzylidene-1-methylpiperidin-4-one* (**4a**). $C_{13}H_{15}NO$; Brown liquid; ^{1}H-NMR (600 MHz, TMS, $CDCl_3$): δ 2.44 (s, 3H), 2.67 (t, *J* = 6.0 Hz, 2H), 2.81 (d, *J* = 6.0 Hz, 2H), 3.65 (s, 2H), 7.34–7.41 (m, 5H), 7.58 (s, 1H); ^{13}C-NMR (150 MHz, $CDCl_3$): δ 39.1, 46.2, 52.8, 57.7, 128.5, 129.1, 130.4, 133.0, 134.9, 135.9, 197.8; MS: *m/z* 202 $[M+H]^+$.

(*E*)-*1-methyl-3-*(*4-nitrobenzylidene*)*piperidin-4-one* (**4b**). $C_{13}H_{14}N_2O_3$; Yellow solid; m.p. 141–142 ℃; ^{1}H-NMR (400 MHz, TMS, $CDCl_3$): δ 2.45 (s, 3H), 2.71 (t, *J* = 6.0 Hz, 2H), 2.85 (t, *J* = 6.0 Hz, 2H),

3.62 (s, 2H), 7.48 (d, *J* = 8.4 Hz, 2H), 7.55 (s, 1H), 8.26 (d, *J* = 8.4 Hz, 2H); ^{13}C-NMR (100 MHz, $CDCl_3$): δ 39.2, 46.2, 52.7, 57.5, 123.7, 130.8, 132.8, 136.0, 141.3, 147.5, 197.3; MS: *m/z* 247 $[M+H]^+$.

(*E*)-*4-((1-methyl-4-oxopiperidin-3-ylidene)methyl)benzonitrile* (**4c**). $C_{14}H_{14}N_2O$; Yellow solid; m.p. 122–123 ℃; ^{1}H-NMR (400 MHz, TMS, $CDCl_3$): δ 2.45 (s, 3H), 2.70 (t, *J* = 6.0 Hz, 2H), 2.84 (t, *J* = 6.0 Hz, 2H), 3.60 (s, 2H), 7.42 (d, *J* = 8.0 Hz, 2H), 7.51 (s, 1H), 7.69 (d, *J* = 8.0 Hz, 2H); ^{13}C-NMR (100 MHz, $CDCl_3$): δ 39.1, 46.1, 52.6, 57.4, 112.3, 118.4, 130.5, 132.2, 133.2, 135.6, 139.4, 197.3; MS: *m/z* 227 $[M+H]^+$.

(*E*)-*3-(4-fluorobenzylidene)-1-methylpiperidin-4-one* (**4d**). $C_{13}H_{14}FNO$; Yellow solid; m.p. 38–39 ℃; ^{1}H-NMR (400 MHz, TMS，$CDCl_3$)：δ 2.45 (s, 3H), 2.67 (t, *J* = 6.0 Hz, 2H), 2.82 (t, *J* = 6.0 Hz, 2H), 3.62 (s, 1H), 3.63 (s, 1H), 7.08–7.12 (m, 2H), 7.32–7.35 (m, 2H), 7.53 (s,1H); ^{13}C-NMR (100 MHz, $CDCl_3$): δ 39.1, 46.2, 52.7, 57.6, 115.7 (d, *J* = 22 Hz), 131.0 (d, *J* = 3 Hz), 132.3 (d, *J* = 8 Hz), 132.7 (d, *J* = 1 Hz), 134.7, 162.9 (d, *J* = 250 Hz), 197.6; MS: *m/z* 220 $[M+H]^+$.

(*E*)-*3-(4-bromobenzylidene)-1-methylpiperidin-4-one* (**4e**). $C_{13}H_{14}BrNO$; Yellow solid; m.p. 63–64 ℃; ^{1}H-NMR (400 MHz, TMS, $CDCl_3$)： δ 2.44 (s, 3H), 2.67 (t, *J* = 6.0 Hz, 2H), 2.82 (t, *J* = 6.0 Hz, 2H), 3.59 (s, 1H), 3.60 (s, 1H), 7.20 (d, *J* =8.4 Hz, 2H), 7.48 (s, 1H), 7.53 (d, *J* = 8.4 Hz, 2H); ^{13}C-NMR (100 MHz, $CDCl_3$): δ 39.1, 46.2, 52.8, 57.7, 123.4, 131.8, 131.8, 133.6, 133.8, 134.5, 197.6; MS: *m/z* 302 $[M+Na]^+$.

(*E*)-*3-(3,4-dichlorobenzylidene)-1-methylpiperidin-4-one* (**4f**). $C_{13}H_{13}Cl_2NO$; Yellow solid; m.p. 73–74 ℃; ^{1}H-NMR (400 MHz, TMS, $CDCl_3$): δ 2.45 (s, 3H), 2.68 (t, *J* = 6.0 Hz, 2H), 2.82 (t, *J* = 6.0 Hz, 2H), 3.59 (s, 1H), 3.60 (s, 1H), 7.15–7.18 (m, 1H), 7.41–7.43 (m, 2H), 7.47–7.49 (m, 1H); ^{13}C-NMR (100 MHz, $CDCl_3$): δ 39.2, 46.2, 52.7, 57.5, 129.4, 130.6, 131.8, 132.9, 133.1, 133.2, 134.5, 134.9, 197.4; MS: *m/z* 270 $[M]^+$.

(*E*)-*1-methyl-3-(4-methylbenzylidene)piperidin-4-one* (**4g**). $C_{14}H_{17}NO$; Brown liquid; ^{1}H-NMR (400 MHz, TMS, $CDCl_3$): δ 2.37 (s, 3H), 2.44 (s, 3H), 2.66 (t, *J* = 6.0 Hz, 2H), 2.80 (t, *J* = 6.0 Hz, 2H), 3.65 (s, 1H), 3.65 (s, 1H), 7.20 (d, *J* = 8.0 Hz, 2H), 7.25 (d, *J* = 8.4 Hz, 2H), 7.56 (s, 1H); ^{13}C-NMR (100 MHz, $CDCl_3$): δ 21.3, 39.0, 46.2, 52.7, 57.8, 129.2, 130.5, 132.0, 132.1, 136.0, 139.3, 197.6; MS: *m/z* 216 $[M+H]^+$.

(*E*)-*3-(4-methoxybenzylidene)-1-methylpiperidin-4-one* (**4h**). $C_{14}H_{17}NO_2$; Yellow solid; m.p. 60–61 ℃; ^{1}H-NMR (400 MHz, TMS, $CDCl_3$): δ 2.46 (s, 3H), 2.66 (t, *J* = 6.0 Hz, 2H), 2.81 (t, *J* = 6.0 Hz, 2H), 3.66 (s, 2H), 3.84 (s, 3H), 6.93 (d, *J* = 8.8 Hz, 2H), 7.33 (d, *J* = 8.4 Hz, 2H), 7.56 (s, 1H); ^{13}C-NMR (100 MHz, $CDCl_3$): δ 39.1, 46.3, 52.7, 55.4, 58.0, 114.1, 127.6, 130.9, 132.3, 132.5, 136.0, 160.4, 197.7. MS: *m/z* 232 $[M+H]^+$.

(*E*)-*3-(3-chlorobenzylidene)-1-methylpiperidin-4-one* (**4i**). $C_{13}H_{14}ClNO$; Yellow solid; m.p. 57–58 ℃; ^{1}H-NMR (400 MHz, TMS, $CDCl_3$): δ 2.45 (s, 3H), 2.68 (t, *J* = 6.0 Hz, 2H), 2.82 (t, *J* = 6.0 Hz, 2H), 3.61 (s, 1H), 3.62 (s, 1H), 7.20–7.22 (m, 1H), 7.31–7.34 (m, 3H), 7.48 (s, 1H); ^{13}C-NMR (100 MHz, $CDCl_3$): δ 39.1, 46.2, 52.7, 57.5, 128.4, 129.0, 129.8, 129.9, 134.1, 134.2, 134.5, 136.7, 197.5; MS: *m/z* 235 $[M+H]^+$.

(*E*)-*3-*(*3-bromobenzylidene*)*-1-methylpiperidin-4-one* (**4j**). $C_{13}H_{14}BrNO$; Yellow solid; m.p. 51–52 °C; ^{1}H-NMR (400 MHz, TMS, $CDCl_3$): δ 2.44 (s, 3H), 2.67 (t, *J* = 6.0 Hz, 2H), 2.82 (t, *J* = 6.0 Hz, 2H), 3.60 (s, 1H), 3.61 (s, 1H), 7.26–7.27 (m, 2H), 7.46–7.48 (m, 3H); ^{13}C-NMR (100 MHz, $CDCl_3$): δ 39.1, 46.1, 52.7, 57.4, 122.5, 128.7, 130.0, 131.8, 132.8, 133.9, 134.2, 136.9, 197.4; MS: *m/z* 302 $[M+Na]^+$.

(*E*)-*3-*(*3-methoxybenzylidene*)*-1-methylpiperidin-4-one* (**4k**). $C_{14}H_{17}NO_2$; Brown liquid; ^{1}H-NMR (400 MHz, TMS, $CDCl_3$): δ 2.44 (s, 3H), 2.67 (t, *J* = 6.0 Hz, 2H), 2.81 (t, *J* = 6.0 Hz, 2H), 3.64 (s, 2H), 3.82 (s, 3H), 6.87–6.95 (m, 3H), 7.30–7.34 (m, 1H), 7.54 (s, 1H); ^{13}C-NMR (100 MHz, $CDCl_3$): δ 39.1, 46.2, 52.8, 55.3, 57.7, 114.6, 115.9, 122.8, 129.5, 133.2, 135.8, 136.2, 159.5, 197.8; MS: *m/z* 232 $[M+H]^+$.

(*E*)-*3-*(*2-fluorobenzylidene*)*-1-methylpiperidin-4-one* (**4l**). $C_{13}H_{14}FNO$; Brown liquid; ^{1}H-NMR (400 MHz, TMS, $CDCl_3$): δ 2.42 (s, 3H), 2.68 (t, *J* = 6.0 Hz, 2H), 2.82 (t, *J* = 6.0 Hz, 2H), 3.52 (s, 2H), 7.08–7.18 (m, 2H), 7.22–7.28 (m, 1H), 7.32–7.37 (m, 1H), 7.61 (s,1H); ^{13}C-NMR (100 MHz, $CDCl_3$): δ 39.2, 46.1, 53.0, 57.5, 115.8 (d, *J* =21 Hz), 122.8, 123.8, 128.3, 130.8 (d, *J* = 24 Hz), 130.9, 135.0, 160.9 (d, *J* =250 Hz), 197.4; MS: *m/z* 220 $[M+H]^+$.

(*E*)-*3-*(*2-bromobenzylidene*)*-1-methylpiperidin-4-one* (**4m**). $C_{13}H_{14}BrNO$; Brown liquid; ^{1}H-NMR (400 MHz, TMS, $CDCl_3$): δ 2.39 (s, 3H), 2.69 (t, *J* = 6.0 Hz, 2H), 2.82 (t, *J* = 6.0 Hz, 2H), 3.46 (s, 2H), 7.16–7.22 (m, 2H), 7.28–7.34 (m, 1H), 7.60–7.63 (m, 2H); ^{13}C-NMR (100 MHz, $CDCl_3$): δ 39.3, 46.0, 53.1, 57.1, 126.8, 127.0, 130.2, 130.4, 133.1, 134.3, 134.8, 135.2, 197.6; MS: *m/z* 302 $[M+Na]^+$.

(*E*)-*1-methyl-3-*(*naphthalen-2-ylmethylene*)*piperidin-4-one* (**4n**). $C_{17}H_{17}NO$; Brown liquid; ^{1}H-NMR (400 MHz, TMS, $CDCl_3$): δ 2.45 (s, 3H), 2.70 (t, *J* = 6.0 Hz, 2H), 2.83 (t, *J* = 6.0 Hz, 2H), 3.74 (s, 1H), 3.75 (s, 1H), 7.43–7.45 (m, 1H), 7.50–7.53 (m, 2H), 7.73 (s, 1H), 7.80–7.87 (m, 4H); ^{13}C-NMR (100 MHz, $CDCl_3$): δ 39.1, 46.2, 52.8, 57.8, 126.6, 127.1, 127.4, 127.7, 128.2, 128.5, 130.5, 132.4, 133.0, 133.2, 133.3, 136.1, 197.7; MS: *m/z* 274 $[M+Na]^+$.

(*E*)-*1-methyl-3-*(*naphthalen-1-ylmethylene*)*piperidin-4-one* (**4o**). $C_{17}H_{17}NO$; Yellow solid; m.p. 62–63 °C; ^{1}H-NMR (400 MHz, TMS, $CDCl_3$): δ 2.35 (s, 3H), 2.74 (t, *J* = 6.0 Hz, 2H), 2.83 (t, *J* = 6.0 Hz, 2H), 3.49 (s, 2H), 7.29–7.30 (m, 1H), 7.45–7.53 (m, 3H), 7.85–7.88 (m, 2H), 7.94–7.96 (m, 1H), 8.13 (s, 1H); ^{13}C-NMR (100 MHz, $CDCl_3$): δ 39.4, 46.0, 53.3, 57.6, 124.7, 124.9, 126.3, 126.6, 126.8, 128.6, 129.4, 131.9, 132.0, 133.5, 134.0, 134.9, 197.8; MS: *m/z* 252 $[M+H]^+$.

(*E*)-*1-methyl-3-*(*pyridin-2-ylmethylene*)*piperidin-4-one* (**4p**). $C_{12}H_{14}N_2O$; Yellow solid; m.p. 120–121 °C; ^{1}H-NMR (400 MHz, TMS, $CDCl_3$): δ 2.48 (s, 3H), 2.70 (t, *J* = 6.0 Hz, 2H), 2.82 (t, *J* = 6.0 Hz, 2H), 4.07 (s, 1H), 4.08 (s, 1H), 7.18–7.21 (m, 1H), 7.40–7.44 (m, 2H), 7.67–7.72 (m, 1H), 8.69–8.70 (m, 1H); ^{13}C-NMR (100 MHz, $CDCl_3$): δ 39.3, 46.2, 52.5, 58.1, 122.7, 127.6, 132.3, 136.2, 136.5, 149.5, 154.6, 198.4; MS: *m/z* 203 $[M+H]^+$.

(*E*)-*1-methyl-3-*(*pyridin-4-ylmethylene*)*piperidin-4-one* (**4q**). $C_{12}H_{14}N_2O$; Brown liquid; ^{1}H-NMR (400 MHz, TMS, $CDCl_3$): δ 2.44 (s, 3H), 2.70 (t, *J* = 6.0 Hz, 2H), 2.84 (t, *J* = 6.0 Hz, 2H), 3.60 (s, 1H), 3.61 (s, 1H), 7.18–7.20 (m, 2H), 7.42–7.43 (m, 1H), 8.65–8.67 (m, 2H); ^{13}C-NMR (100 MHz, $CDCl_3$): δ 39.2, 46.1, 52.7, 57.3, 123.9, 132.3, 136.5, 142.3, 150.1, 197.3; MS: *m/z* 203 $[M+H]^+$.

(*E*)-*1-methyl-3-*(*thiophen-2-ylmethylene*)*piperidin-4-one* (**4r**). $C_{11}H_{13}NOS$; Brown liquid; ^{1}H-NMR (400 MHz, TMS, $CDCl_3$): δ 2.52 (s, 3H), 2.66 (t, *J* = 6.0 Hz, 2H), 2.81 (t, *J* = 6.0 Hz, 2H), 3.68 (s, 1H), 3.69 (s, 1H), 7.13–7.16 (m, 1H), 7.32–7.33 (m, 1H), 7.56–7.57 (m, 1H), 7.78–7.80 (m, 1H); ^{13}C-NMR (100 MHz, $CDCl_3$): δ 39.0, 46.4, 52.3, 57.8, 128.0, 128.4, 129.5, 130.8, 133.5, 138.3, 196.9; MS: *m/z* 208 $[M+H]^+$.

(*E*)-*3-butylidene-1-methylpiperidin-4-one* (**4s**). $C_{10}H_{17}NO$; Brown liquid; ^{1}H-NMR (400 MHz, TMS, $CDCl_3$): δ 0.94 (t, *J* =7.2 Hz, 3H), 1.47–1.52 (m, 2H), 2.06–2.07 (m, 2H), 2.44 (s, 3H), 2.56 (t, *J* = 6.0 Hz, 2H), 2.73 (t, *J* = 6.0 Hz, 2H), 3.33 (s, 2H), 6.68–6.73 (m, 1H); ^{13}C-NMR (100 MHz, $CDCl_3$): δ 13.9, 21.6, 29.7, 39.0, 46.2, 52.8, 56.0, 133.1, 140.0, 197.2; MS: *m/z* 168 $[M+H]^+$.

(*E*)-*tert-butyl-3-benzylidene-4-oxopiperidine-1-carboxylate* (**4t**). $C_{17}H_{21}NO_3$; Yellow solid; m.p. 107–108 °C; ^{1}H-NMR (400 MHz, TMS, $CDCl_3$): δ 1.44 (s, 9H), 2.66 (t, *J* = 6.0 Hz, 2H), 3.78 (t, *J* = 6.0 Hz, 2H), 4.69 (s, 2H), 7.37–7.42 (m, 5H), 7.63 (s, 1H); ^{13}CNMR (100 MHz, $CDCl_3$): 28.3, 39.1, 40.9, 44.9, 80.5, 128.7, 129.5, 130.5, 131.8, 134.4, 137.2, 154.5, 197.4; MS: *m/z* 310 $[M+Na]^+$.

(*E*)-*3-benzylidenedihydro-2H-pyran-4*(*3H*)*-one* (**4u**). $C_{12}H_{12}O_2$; Yellow solid; m.p. 96–97 °C; ^{1}H-NMR (600 MHz, TMS, $CDCl_3$): δ 2.70 (t, *J* = 6.0 Hz, 2H), 4.09 (t, *J* = 6.0 Hz, 2H), 4.87 (s, 2H), 7.28–7.30 (m, 2H), 7.38–7.43 (m, 3H), 7.64 (s, 1H); ^{13}C-NMR (150 MHz, $CDCl_3$): δ 39.8, 65.6, 68.7, 128.7, 129.5, 130.6, 133.3, 134.3, 136.2, 196.2; MS: *m/z* 211 $[M+Na]^+$.

(*E*)-*3-*(*4-nitrobenzylidene*)*dihydro-2H-pyran-4*(*3H*)*-one* (**4v**). $C_{12}H_{11}NO_4$; Yellow solid; m.p. 197–198 °C; ^{1}H-NMR (400 MHz, TMS, $CDCl_3$): δ 2.74 (t, *J* = 6.0 Hz, 2H), 4.11 (t, *J* = 6.0 Hz, 2H), 4.83 (s, 1H), 4.84 (s, 1H), 7.43 (d, *J* = 8.8 Hz, 2H), 7.62 (s, 1H), 8.27 (d, *J* = 8.8 Hz, 2H); ^{13}C-NMR (100 MHz, $CDCl_3$): δ 39.9, 65.6, 68.4, 123.9, 130.9, 132.9, 136.4, 140.7, 147.8, 195.5; MS: *m/z* 256 $[M+Na]^+$.

(*E*)-*2-*(*4-nitrobenzylidene*)*cyclohexanone* (**4w**). $C_{13}H_{13}NO_3$; Yellow solid; m.p. 119–120 °C; ^{1}H-NMR(400 MHz, TMS, $CDCl_3$): δ 1.78–1.84 (m, 2H), 1.94–2.00 (m, 2H), 2.58 (t, *J* = 6.8 Hz, 2H), 2.82 (t, *J* = 6.8 Hz, 2H), 7.46 (s, 1H), 7.52 (d, *J* = 8.8 Hz, 2H), 8.24 (d, *J* = 8.8 Hz, 2H); ^{13}C-NMR (100 MHz, $CDCl_3$): δ 23.3, 23.8, 29.1, 40.5, 123.6, 130.7, 132.5, 140.0, 142.2, 147.3, 201.2; MS: *m/z* 232 $[M+H]^+$.

(*E*)-*2-*(*4-nitrobenzylidene*)*cyclopentanone* (**4x**). $C_{12}H_{11}NO_3$; Yellow solid; m.p. 139–140 °C; ^{1}H-NMR (400 MHz, TMS, $CDCl_3$): δ 2.05–2.13 (m, 2H), 2.45–2.49 (m, 2H), 3.00–3.03 (m, 2H), 7.39–7.40 (m, 1H), 7.67 (d, *J* = 8.8 Hz, 2H), 8.27 (d, *J* = 8.8 Hz, 2H); ^{13}C-NMR (100 MHz, $CDCl_3$): δ 20.1, 29.4, 37.7, 123.9, 129.3, 130.8, 139.9, 142.0, 147.6, 207.3; MS: *m/z* 240 $[M+Na]^+$.

(**D**) Copies of NMR Spectra for Mono-Arylidene Derivatives

Figure S1. The ^{1}H and ^{13}C-NMR spectra of **4a–c**. (**A-1**) ^{1}H-NMR spectrum of compound **4a**; (**A-2**) ^{13}C-NMR spectrum of compound **4a**; (**B-1**) ^{1}H-NMR spectrum of compound **4b**; (**B-2**) ^{13}C-NMR spectrum of compound **4b**; (**C-1**) ^{1}H-NMR spectrum of compound **4c**; (**C-2**) ^{13}C-NMR spectrum of compound **4c**.

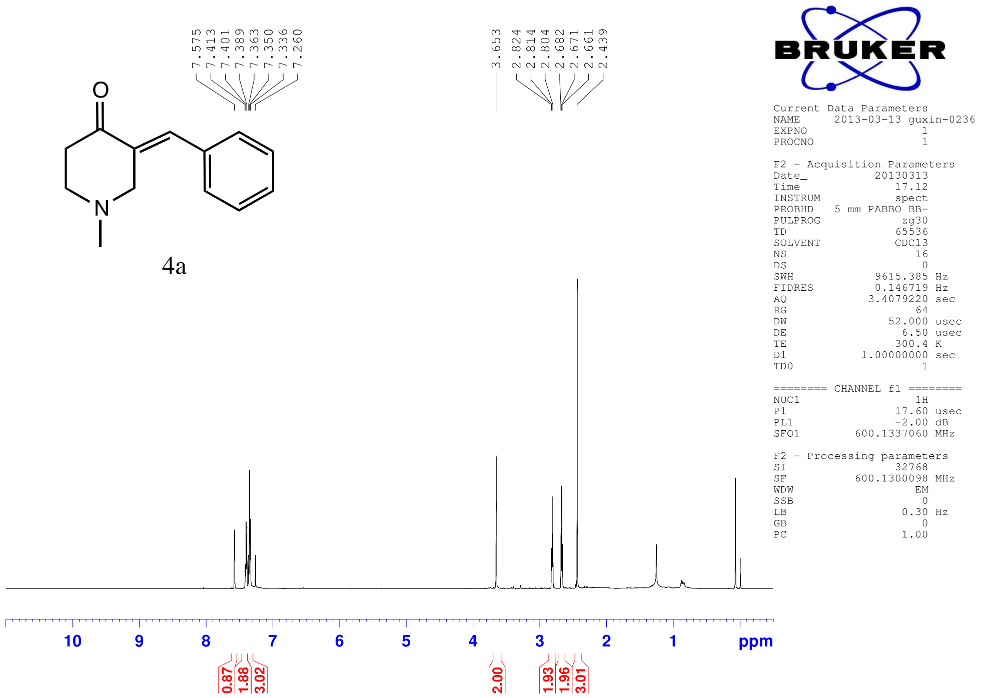

(A-1)

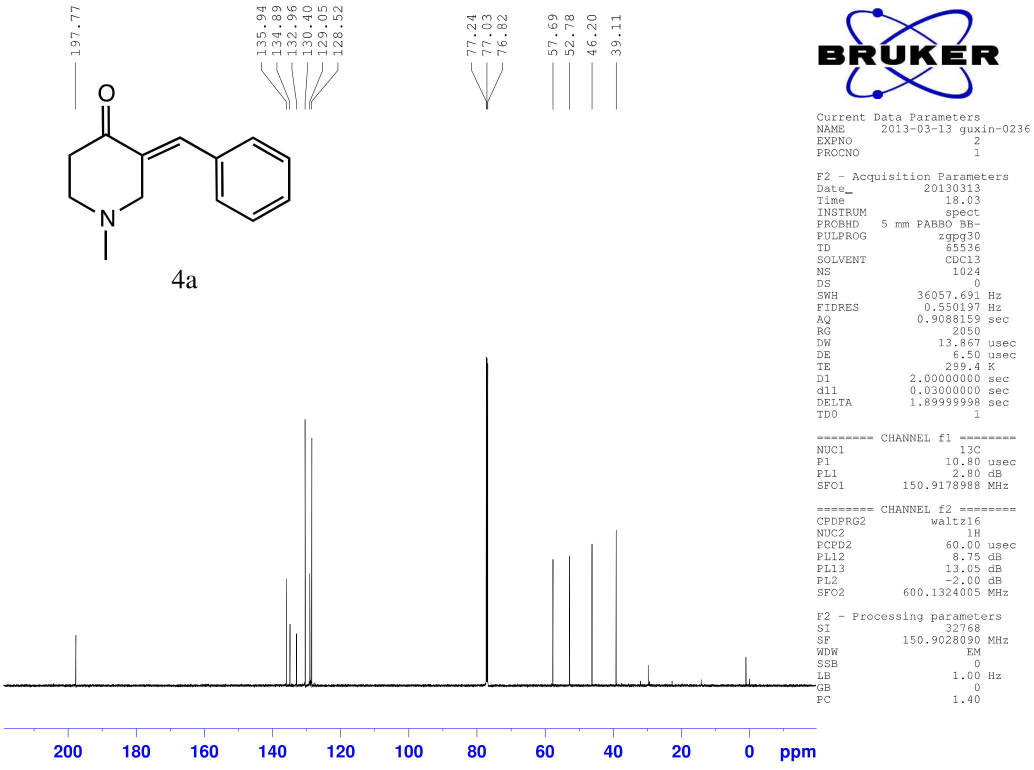

(A-2)

Figure S1. *Cont.*

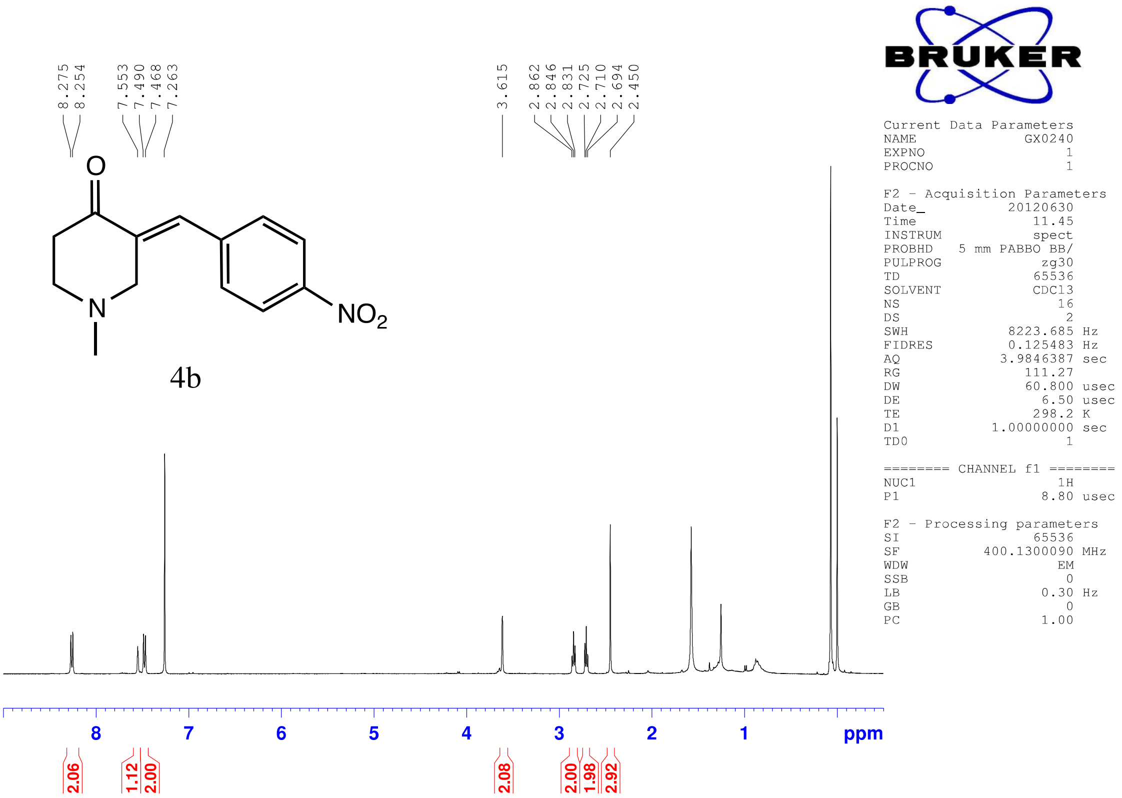

(B-1)

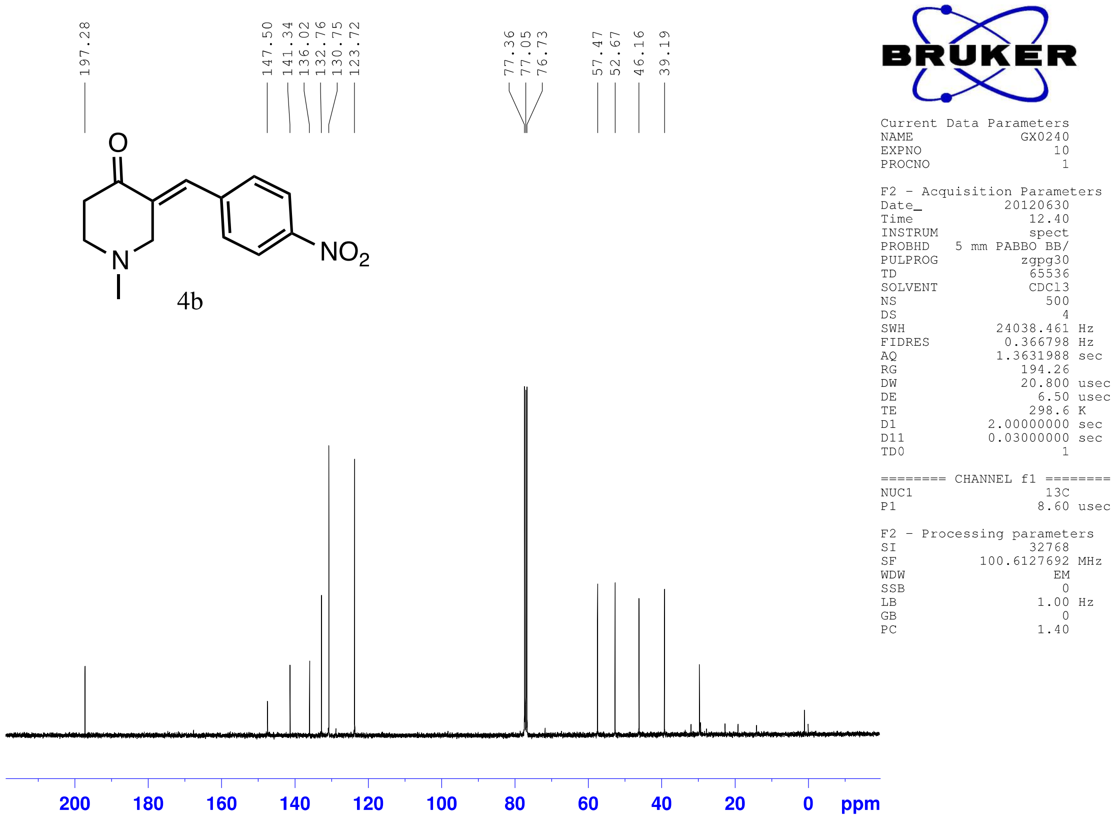

(B-2)

Figure S1. *Cont.*

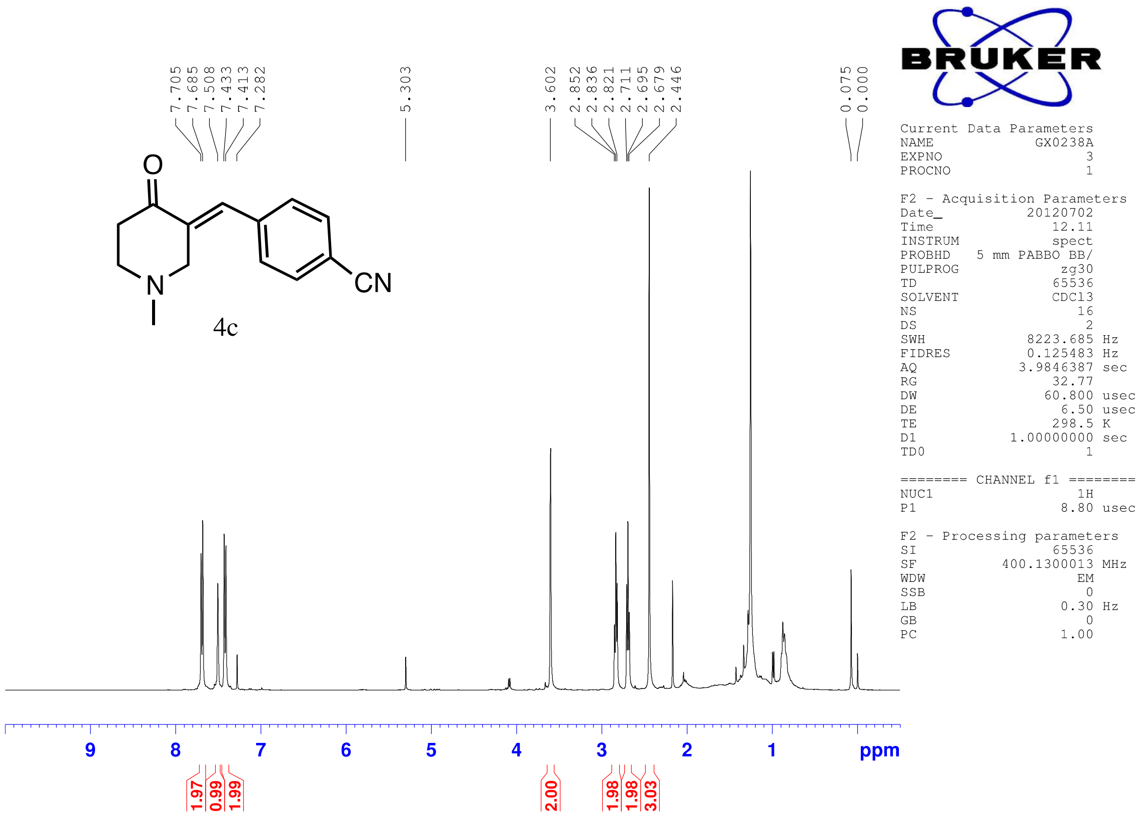

(C-1)

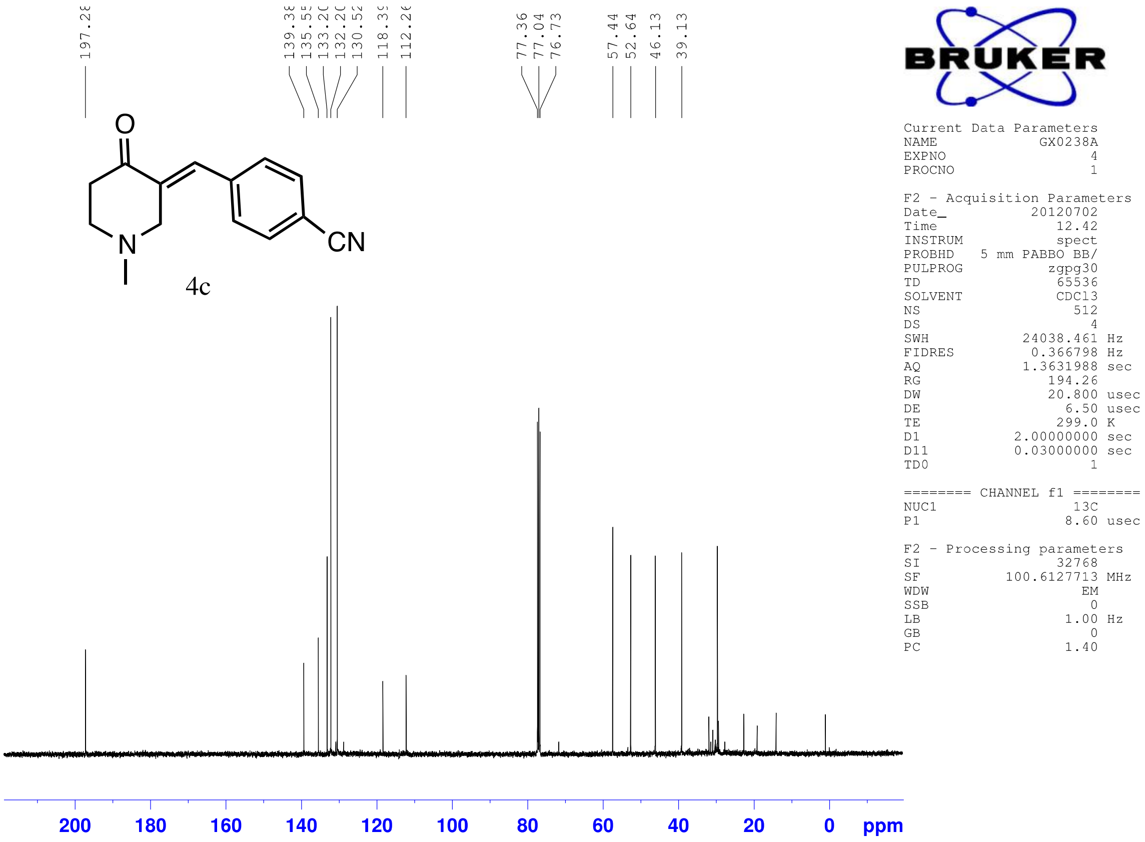

(C-2)

Figure S2. The ^{1}H and ^{13}C-NMR spectra of **4d–f**. (**D-1**) ^{1}H-NMR spectrum of compound **4d**; (**D-2**) ^{13}C-NMR spectrum of compound **4d**; (**E-1**) ^{1}H-NMR spectrum of compound **4e**; (**E-2**) ^{13}C-NMR spectrum of compound **4e**; (**F-1**) ^{1}H-NMR spectrum of compound **4f**; (**F-2**) ^{13}C-NMR spectrum of compound **4f**.

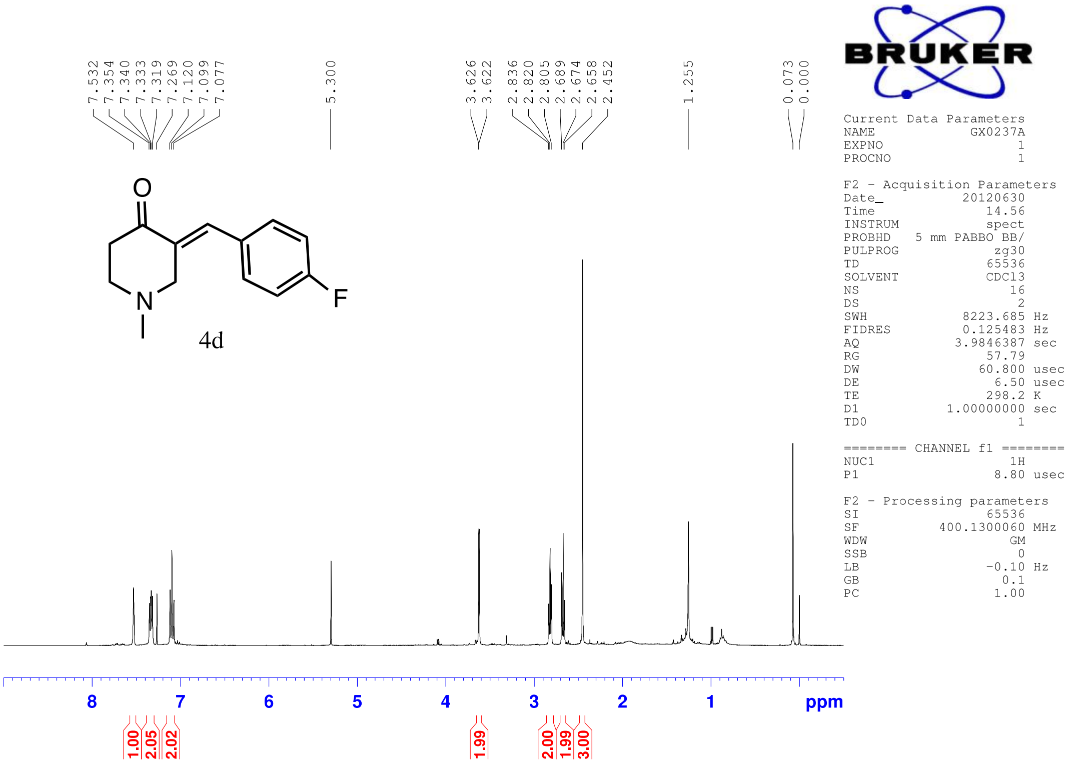

(**D-1**)

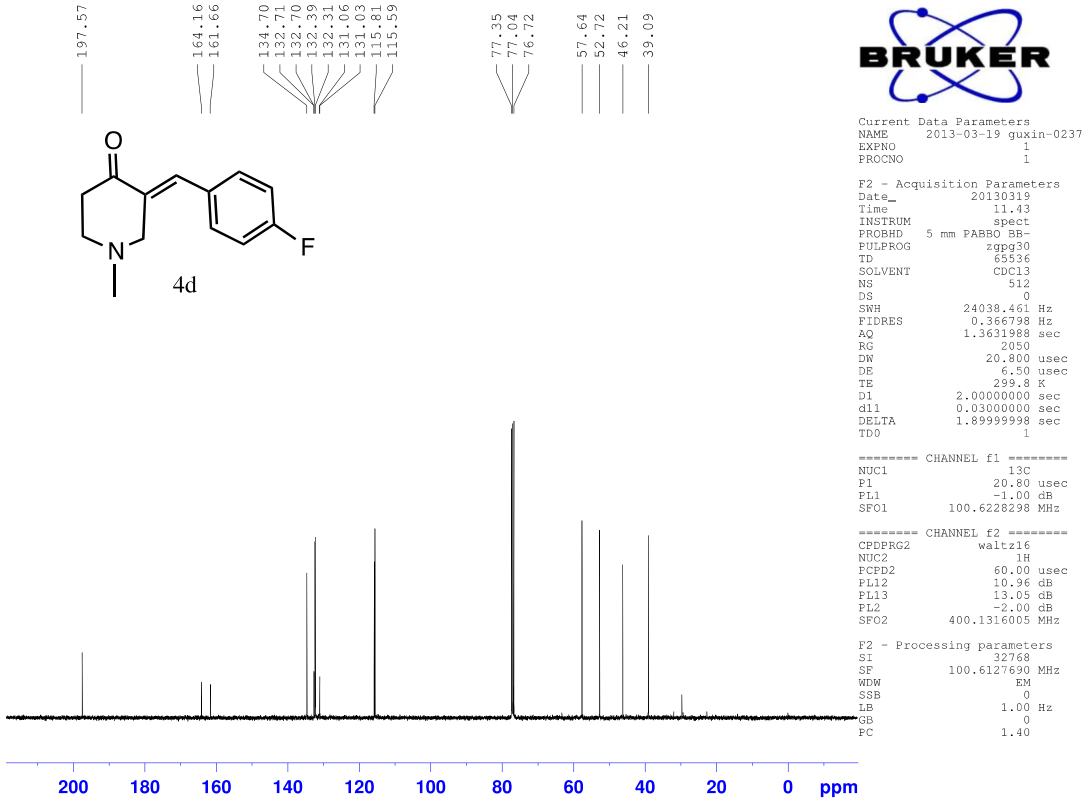

(**D-2**)

Figure S2. *Cont.*

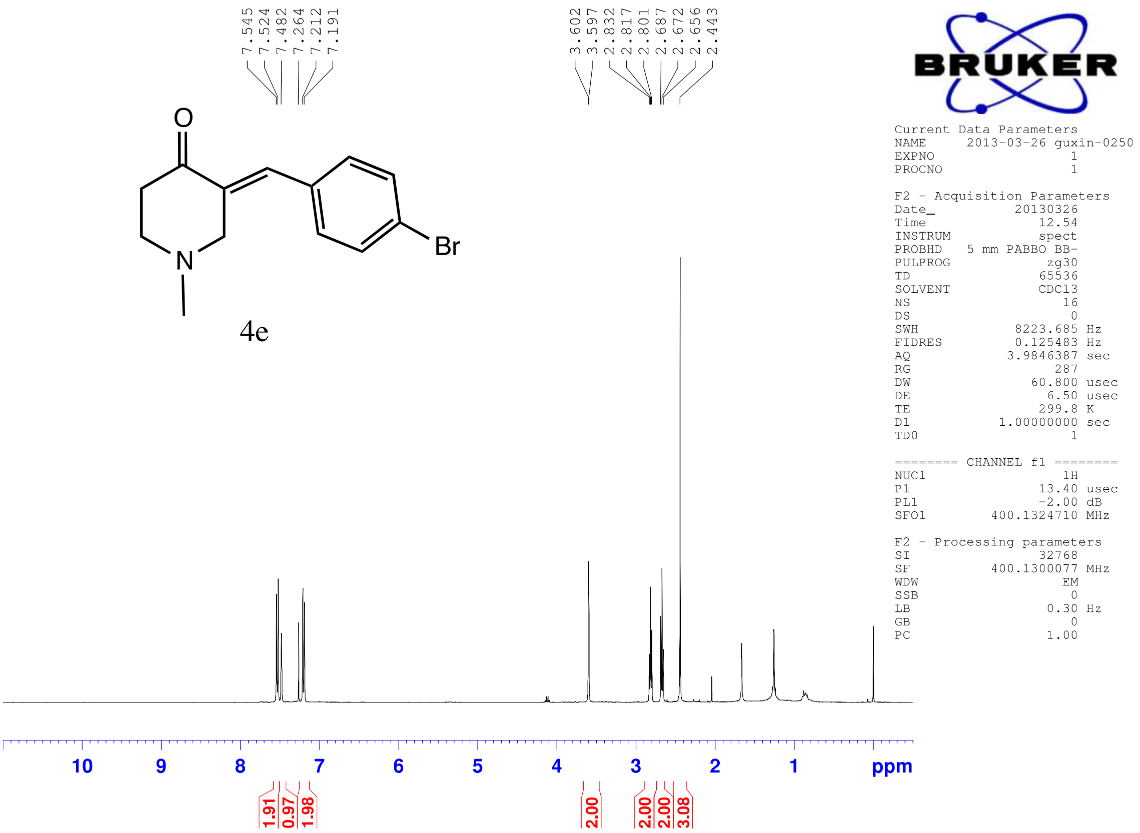

(E-1)

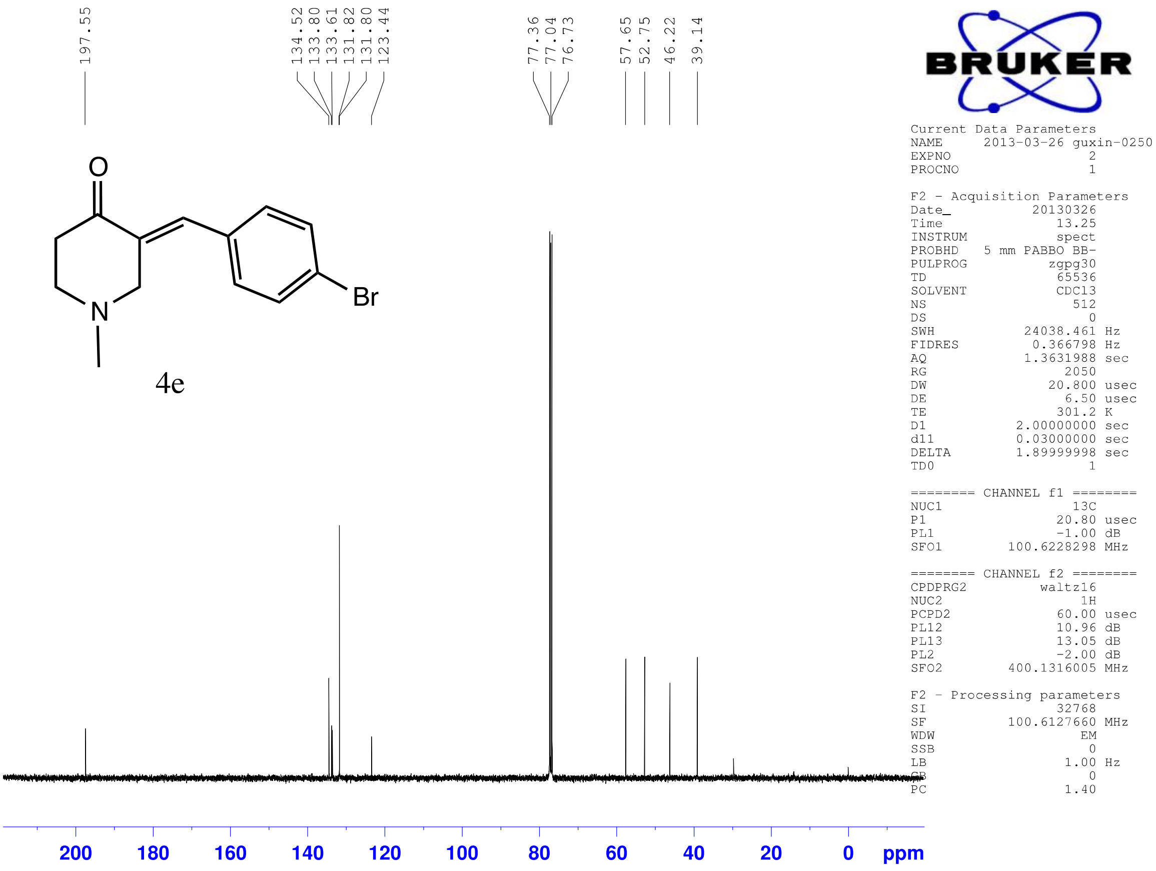

(E-2)

Figure S2. *Cont.*

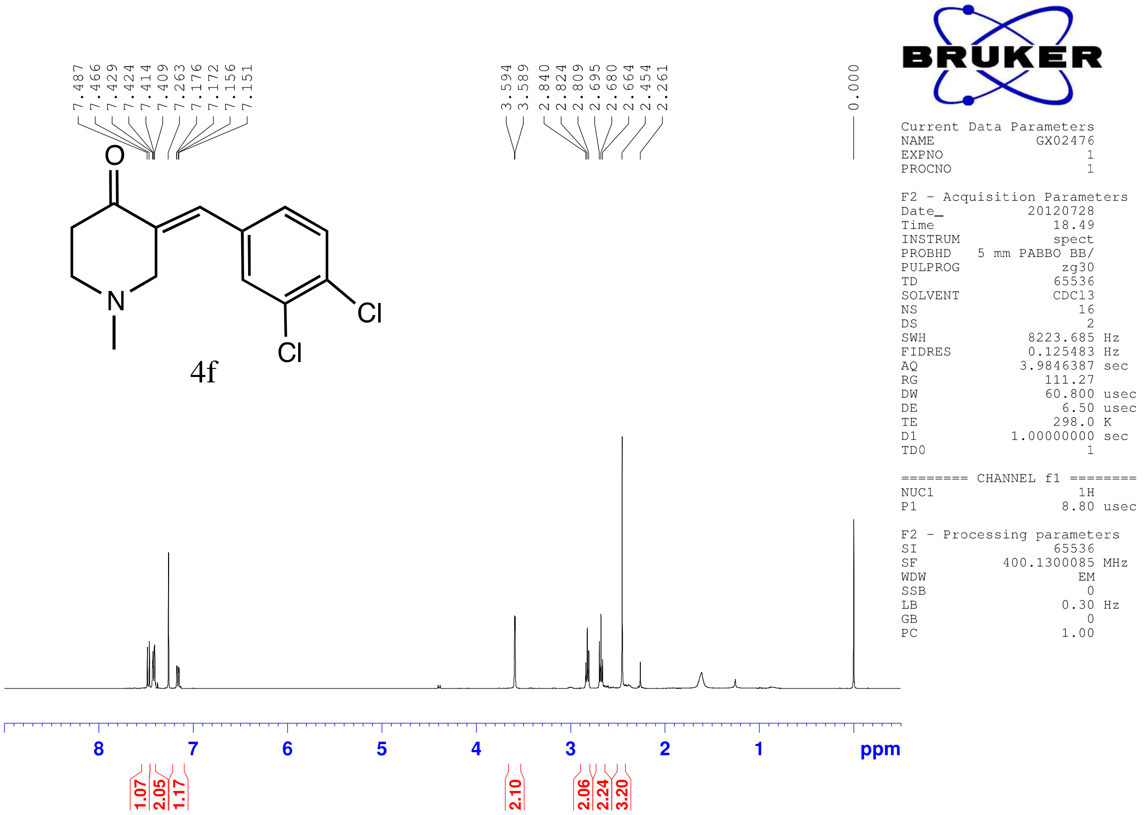

(F-1)

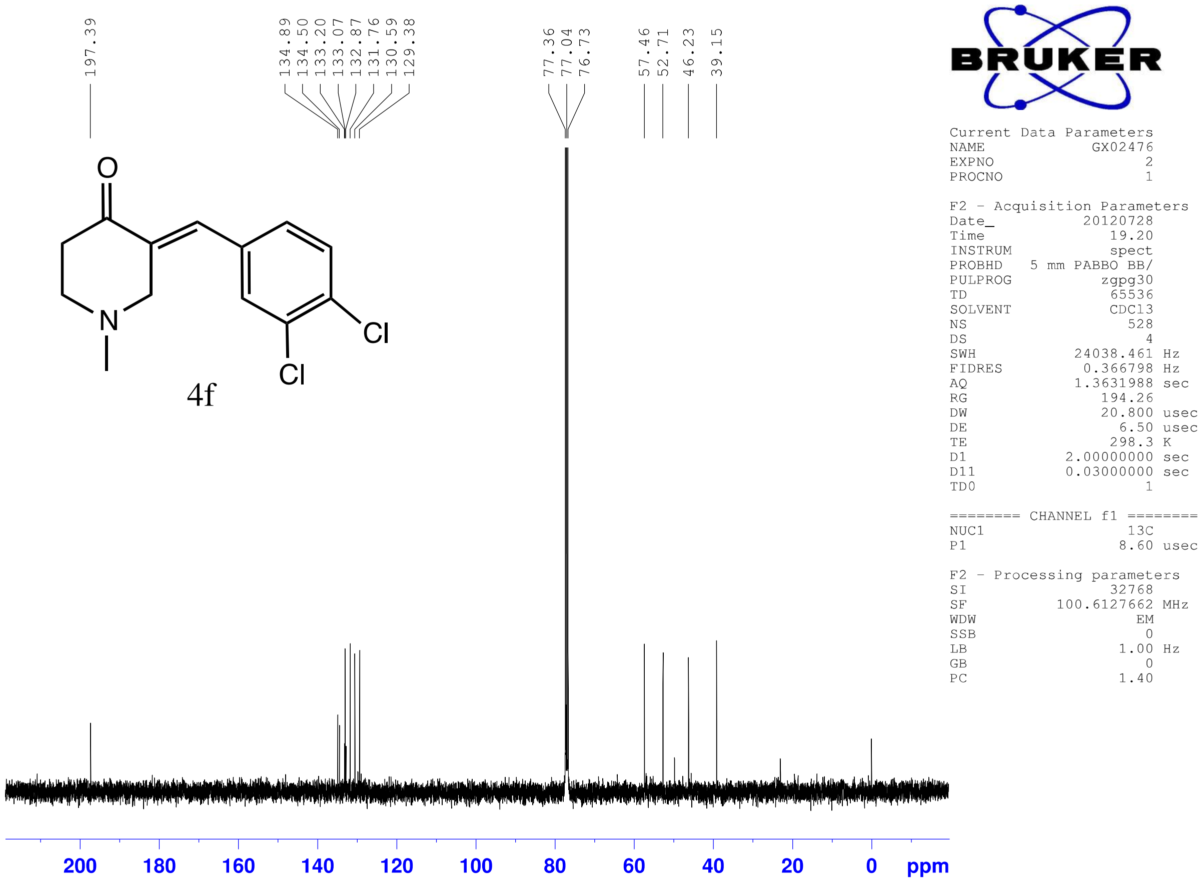

(F-2)

Figure S3. The ^{1}H and ^{13}C-NMR spectra of **4g–i**. (**G-1**) ^{1}H-NMR spectrum of compound **4g**; (**G-2**) ^{13}C-NMR spectrum of compound **4g**; (**H-1**) ^{1}H-NMR spectrum of compound **4h**; (**H-2**) ^{13}C-NMR spectrum of compound **4h**; (**I-1**) ^{1}H-NMR spectrum of compound **4i**; (**I-2**) ^{13}C-NMR spectrum of compound **4i**.

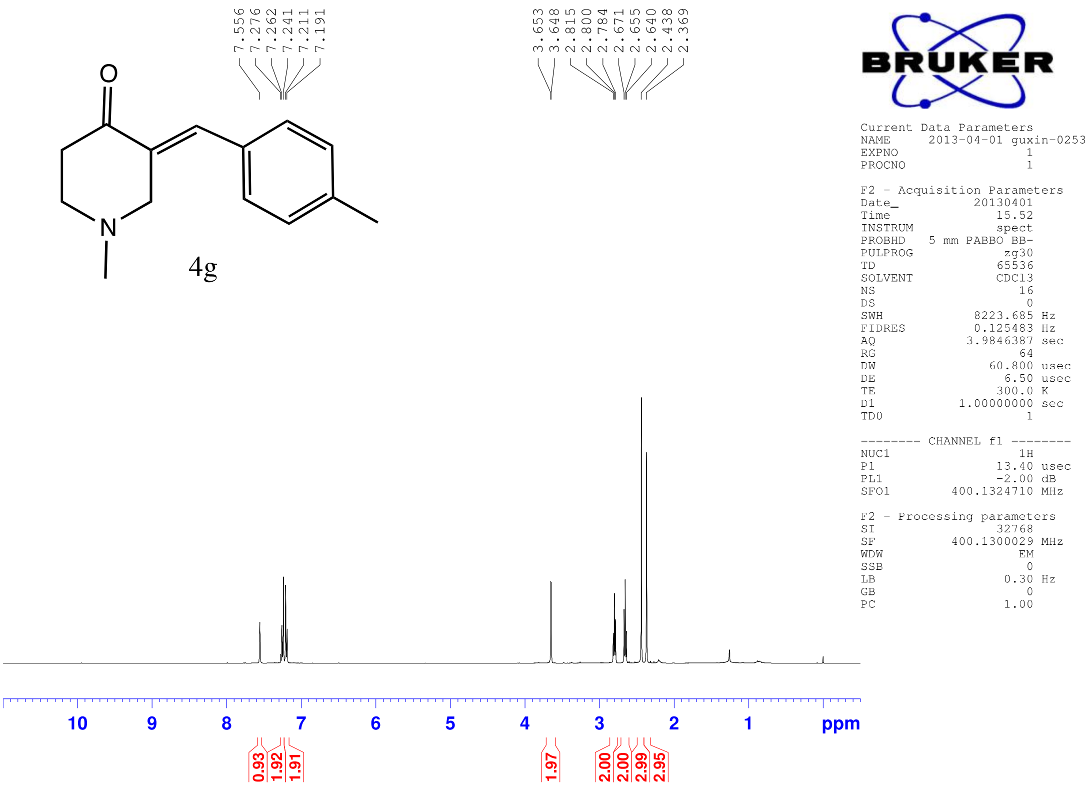

(G-1)

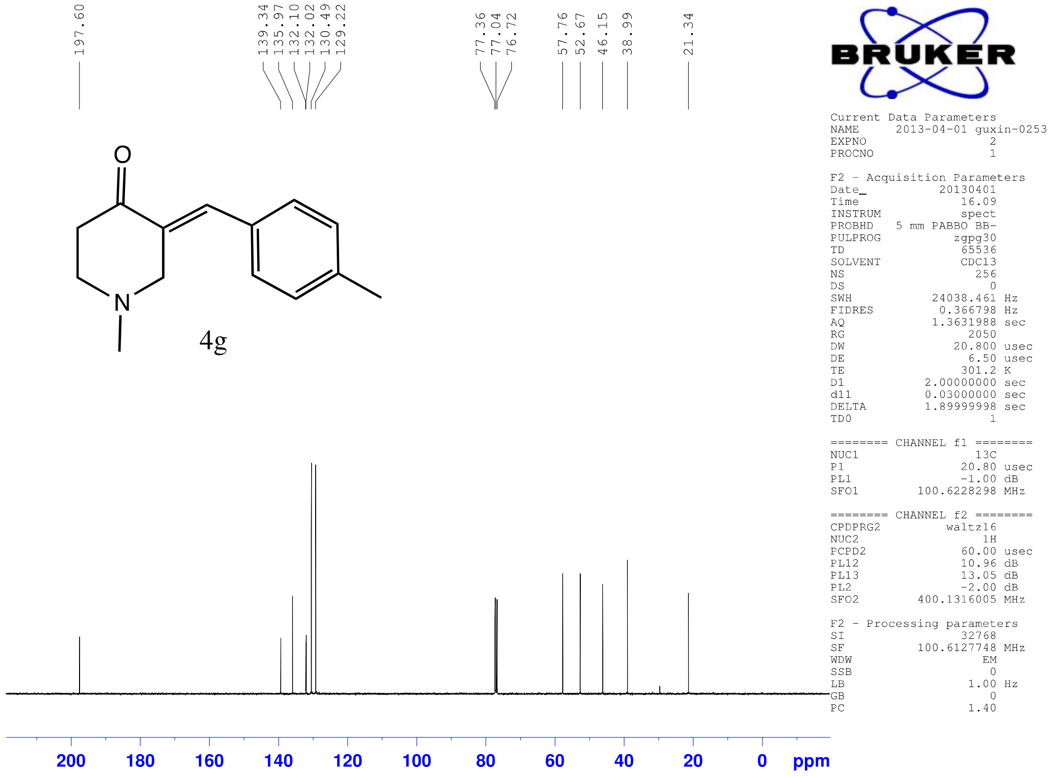

(G-2)

Figure S3. *Cont.*

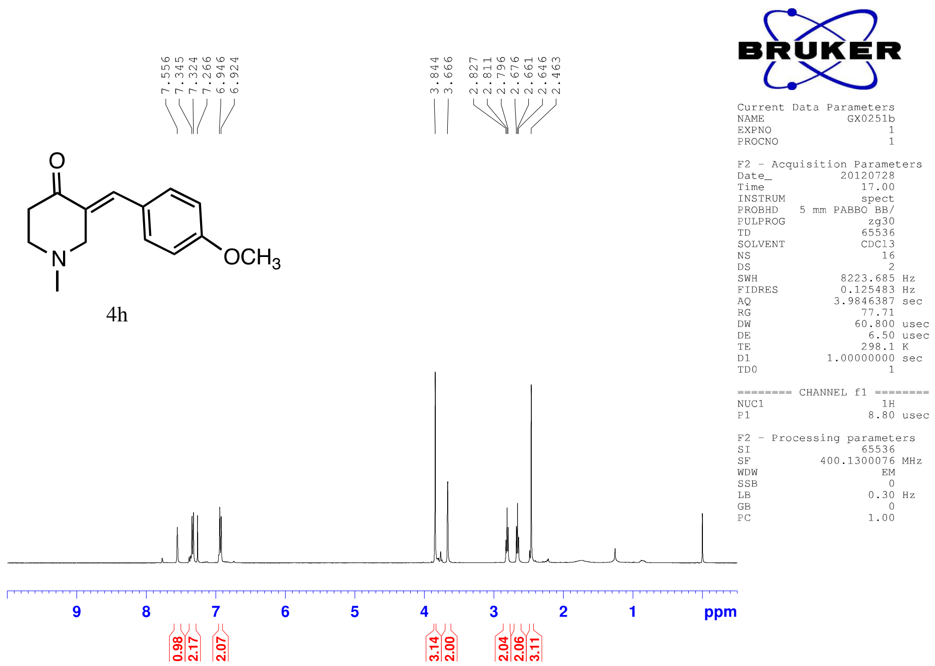

(H-1)

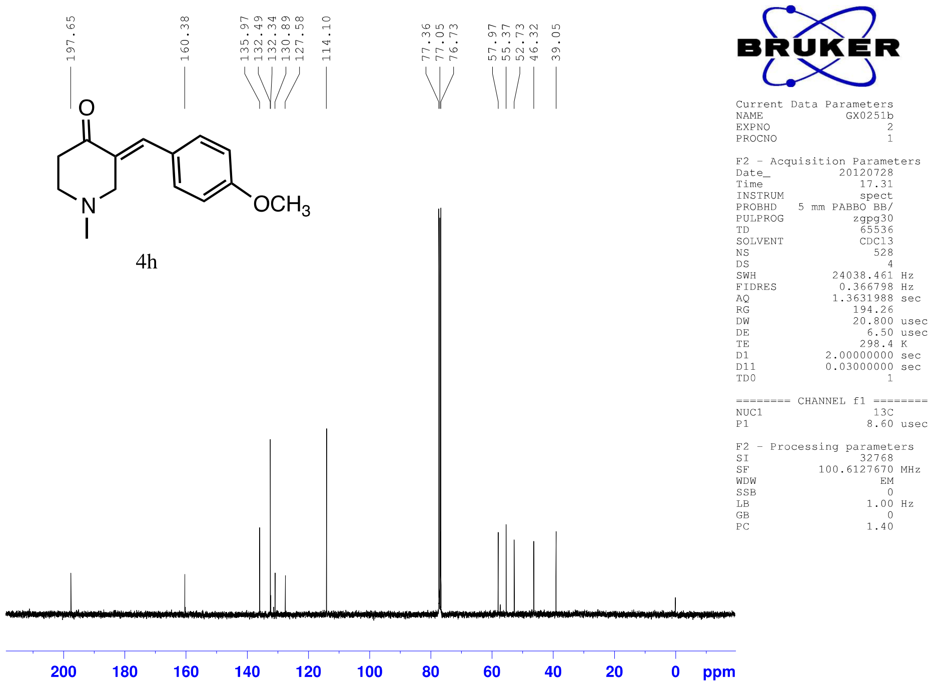

(H-2)

Figure S3. *Cont.*

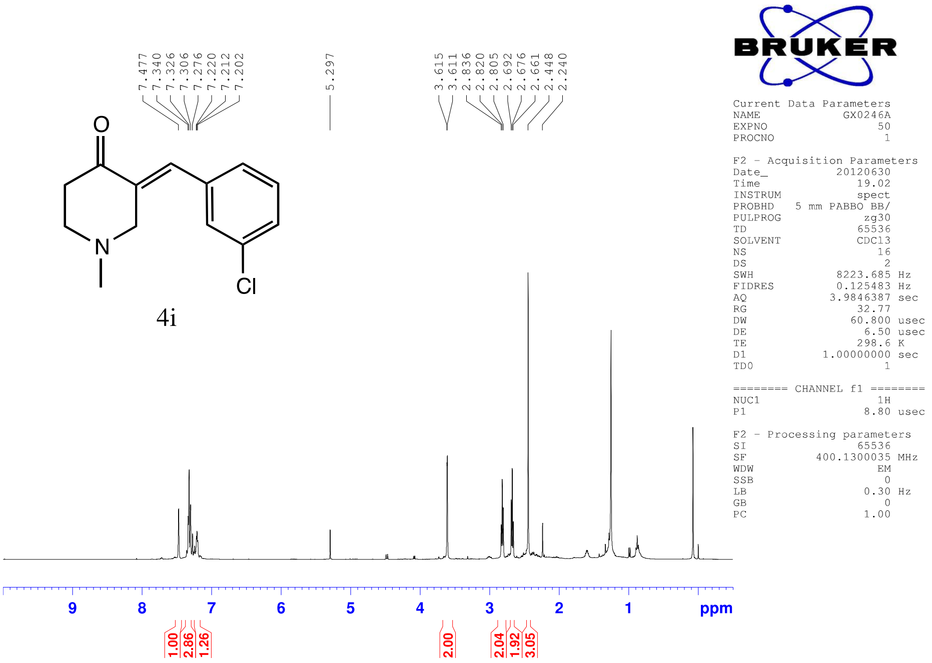

(I-1)

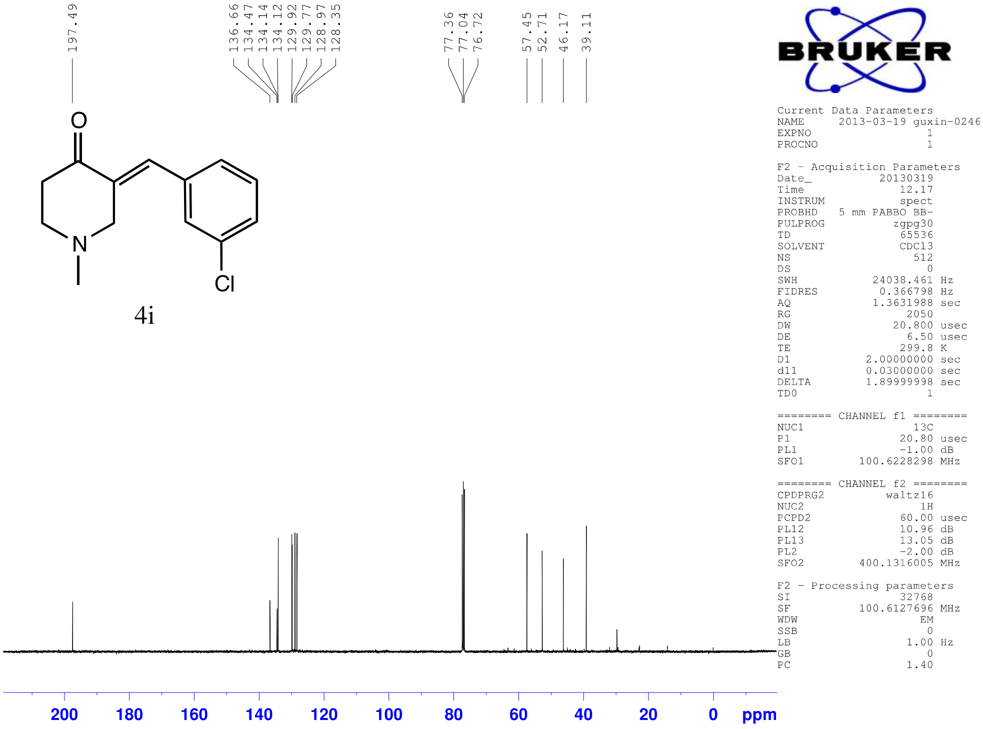

(I-2)

Figure S4. The ^{1}H and ^{13}C-NMR spectra of **4j–l**. (**J-1**) ^{1}H-NMR spectrum of compound **4j**; (**J-2**) ^{13}C-NMR spectrum of compound **4j**; (**K-1**) ^{1}H-NMR spectrum of compound **4k**; (**K-2**) ^{13}C-NMR spectrum of compound **4k**; (**L-1**) ^{1}H-NMR spectrum of compound **4l**; (**L-2**) ^{13}C-NMR spectrum of compound **4l**.

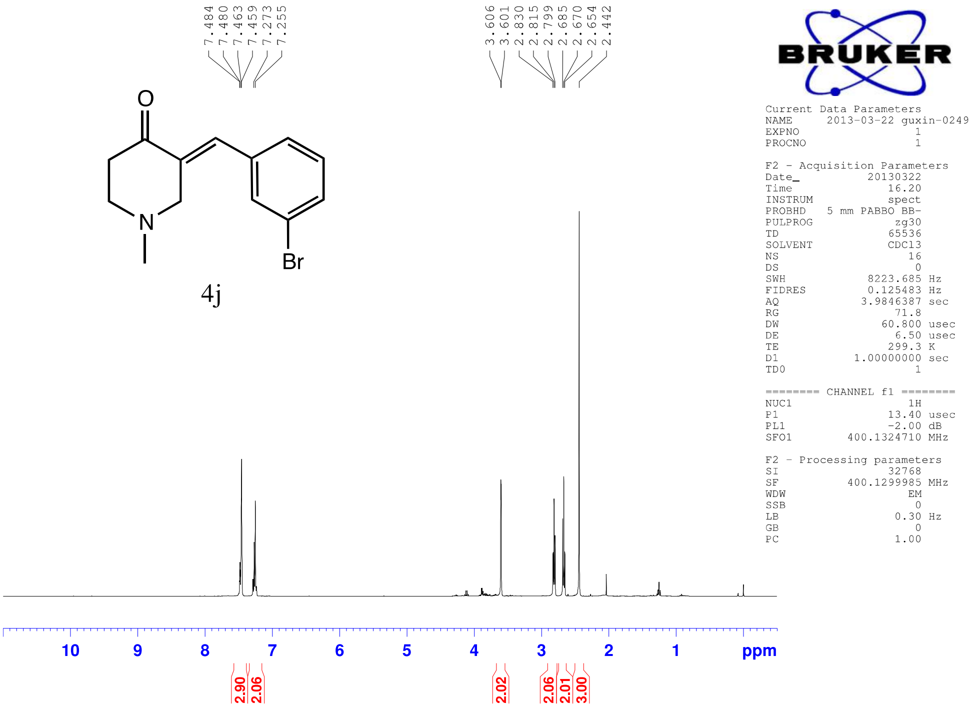

(J-1)

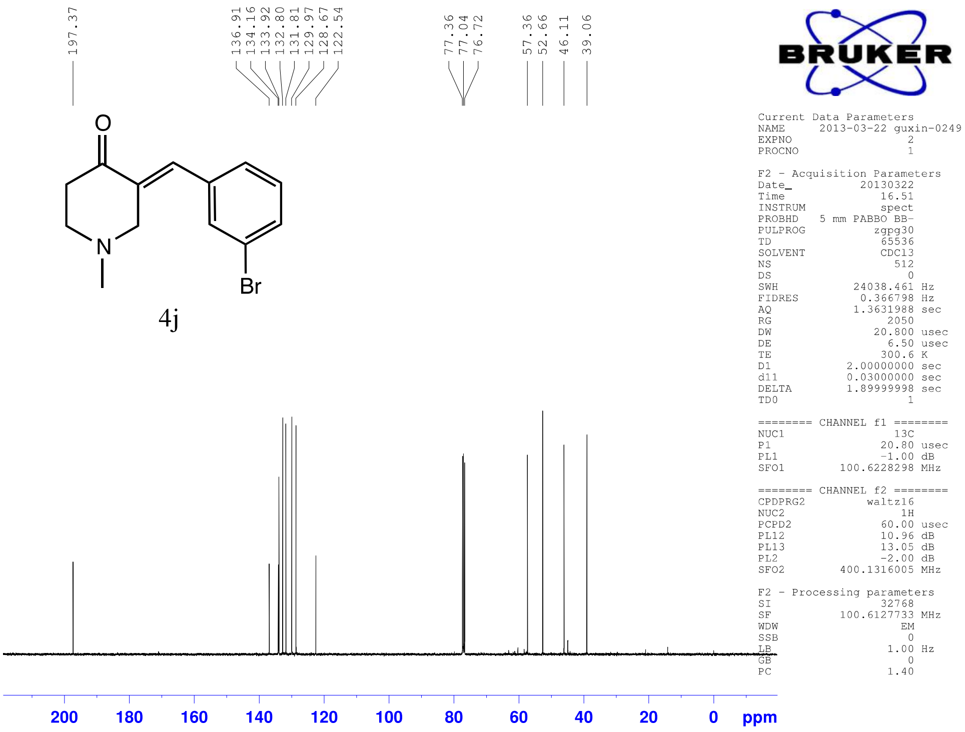

(J-2)

Figure S4. *Cont.*

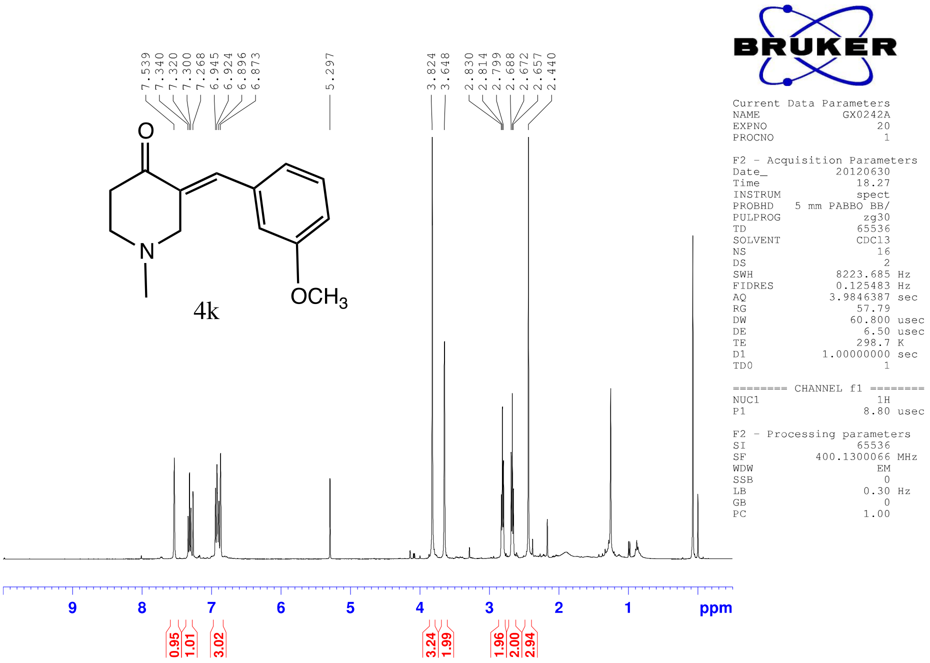

(K-1)

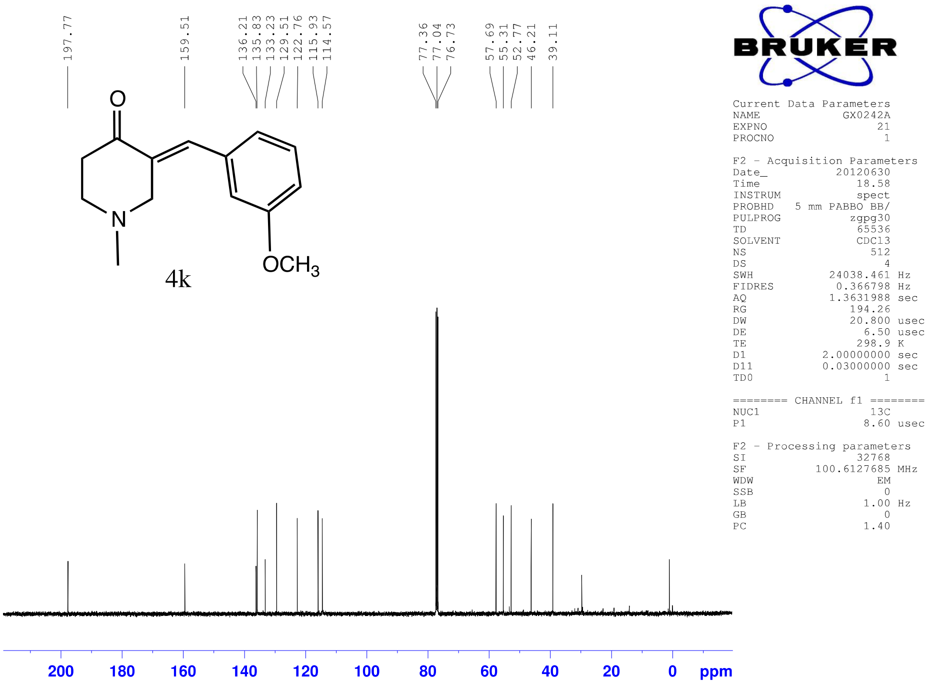

(K-2)

Figure S4. *Cont.*

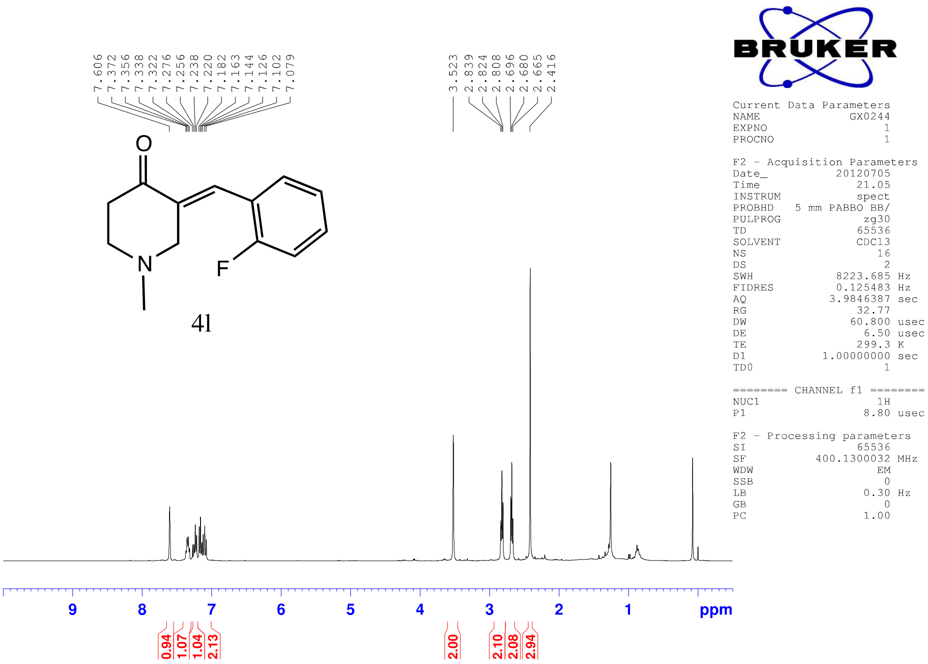

(L-1)

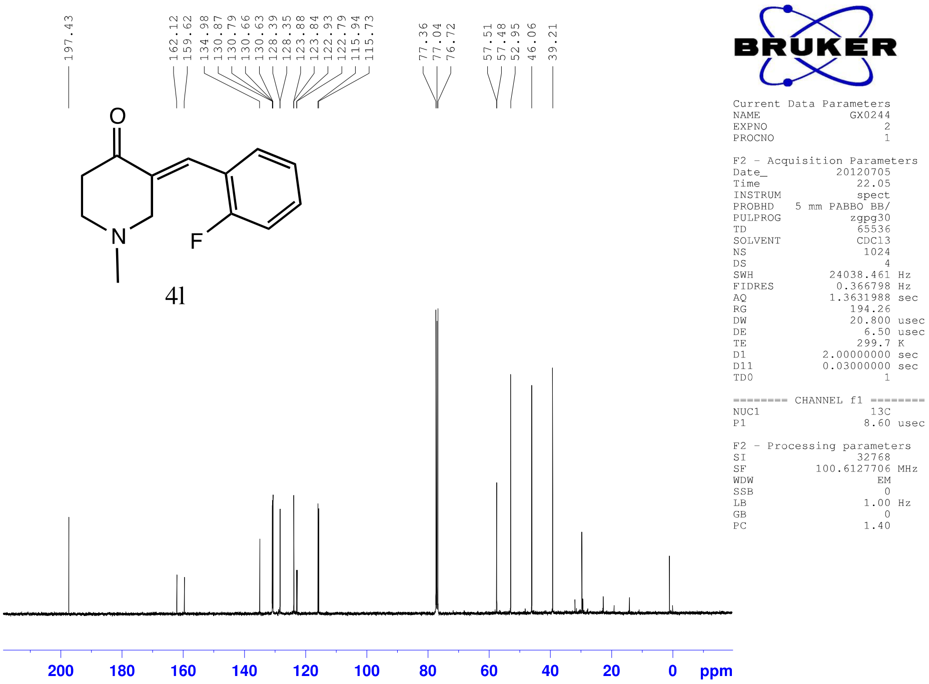

(L-2)

Figure S5. The ^{1}H and ^{13}C-NMR spectra of **4m–o**. (**M-1**) ^{1}H-NMR spectrum of compound **4m**; (**M-2**) ^{13}C-NMR spectrum of compound **4m**; (**N-1**) ^{1}H-NMR spectrum of compound **4n**; (**N-2**) ^{13}C-NMR spectrum of compound **4n**; (**O-1**) ^{1}H-NMR spectrum of compound **4o**; (**O-2**) ^{13}C-NMR spectrum of compound **4o**.

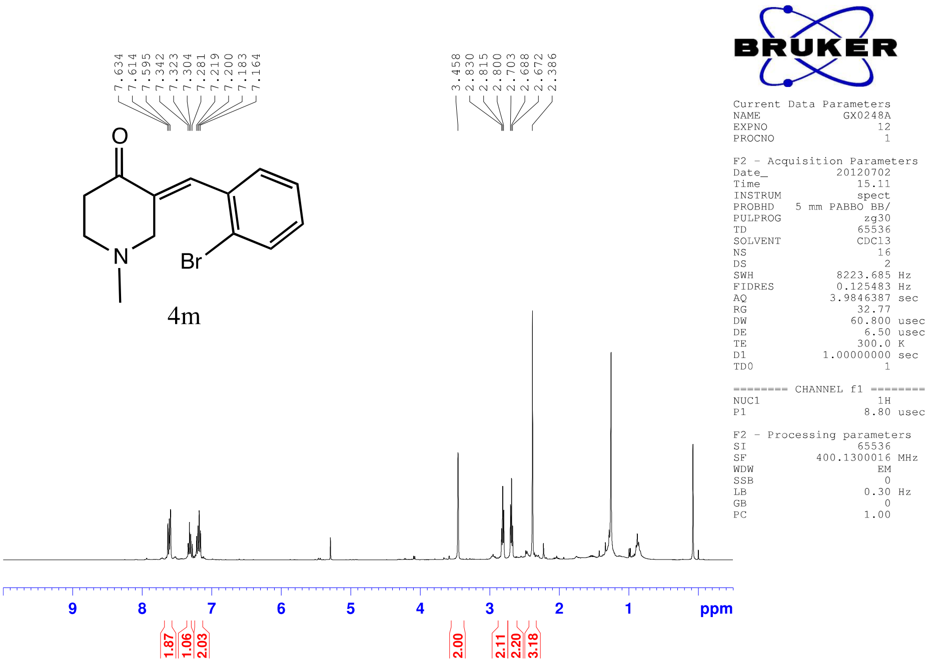

(**M-1**)

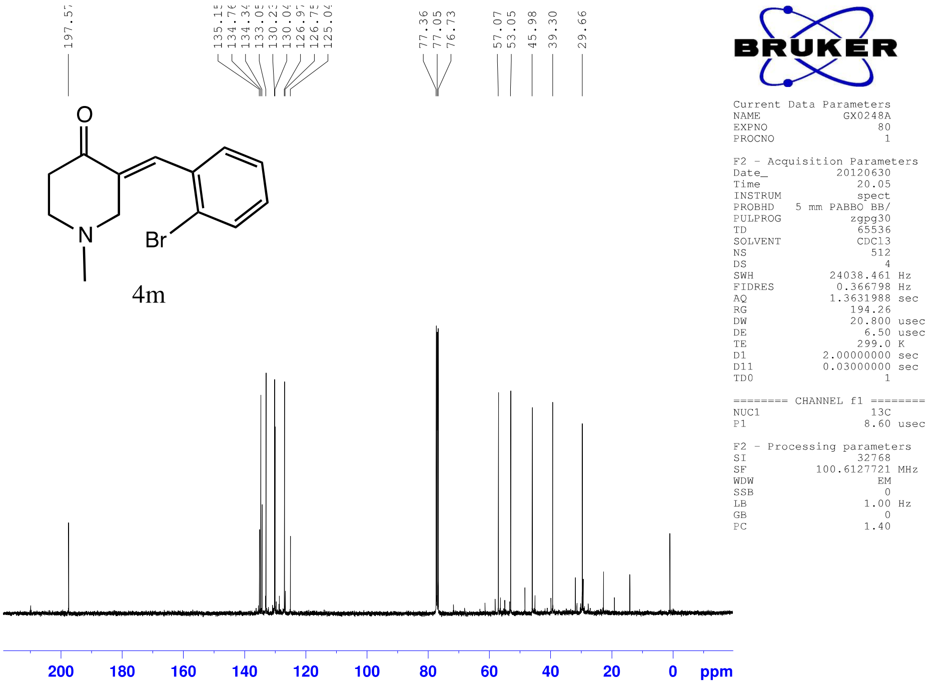

(**M-2**)

Figure S5. *Cont.*

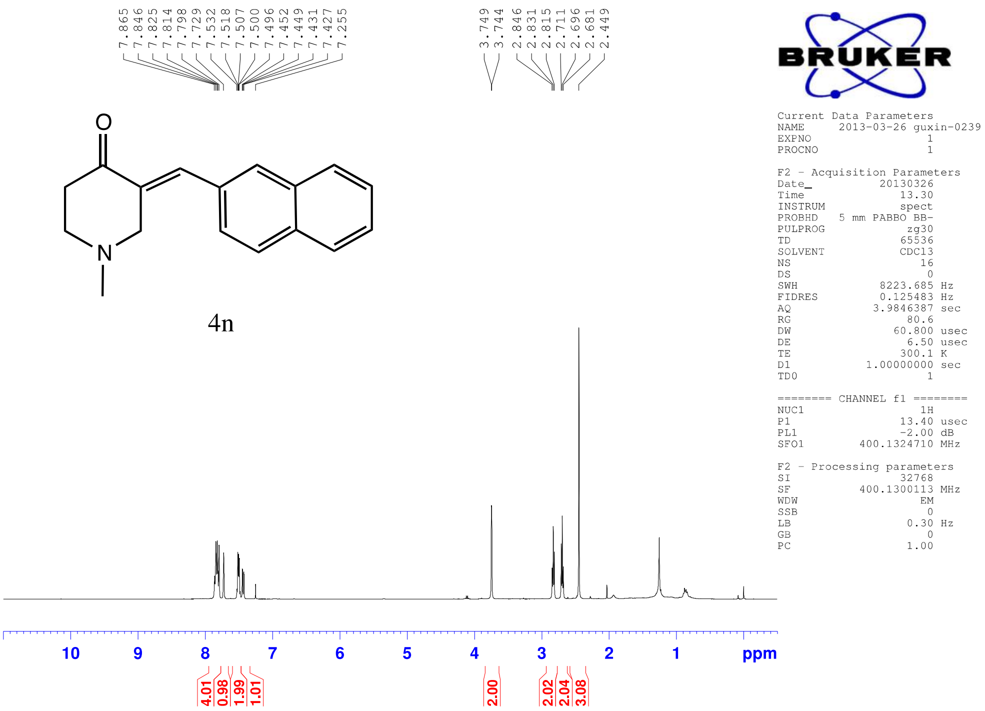

(N-1)

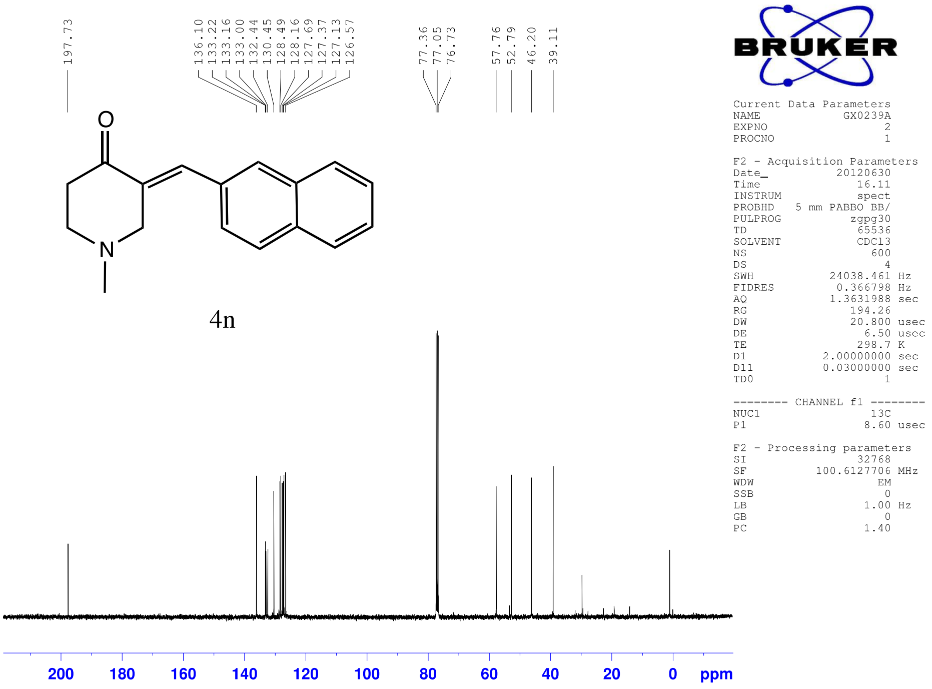

(N-2)

Figure S5. *Cont.*

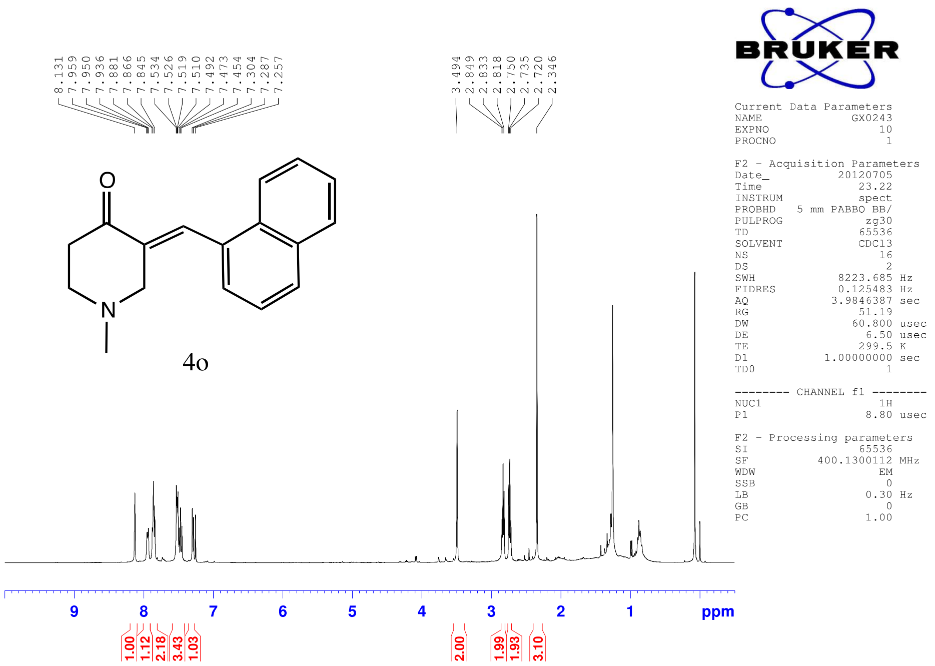

(O-1)

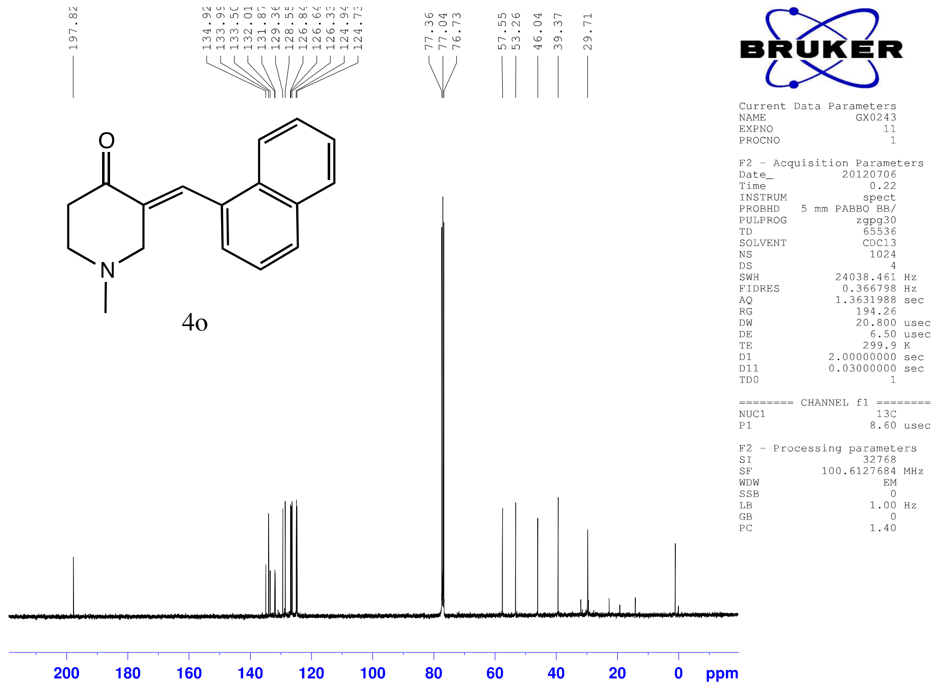

(O-2)

Figure S6. The ^{1}H and ^{13}C-NMR spectra of **4p–q**. (**P-1**) ^{1}H-NMR spectrum of compound **4p**; (**P-2**) ^{13}C-NMR spectrum of compound **4p**; (**Q-1**) ^{1}H-NMR spectrum of compound **4q**; (**Q-2**) ^{13}C-NMR spectrum of compound **4q**.

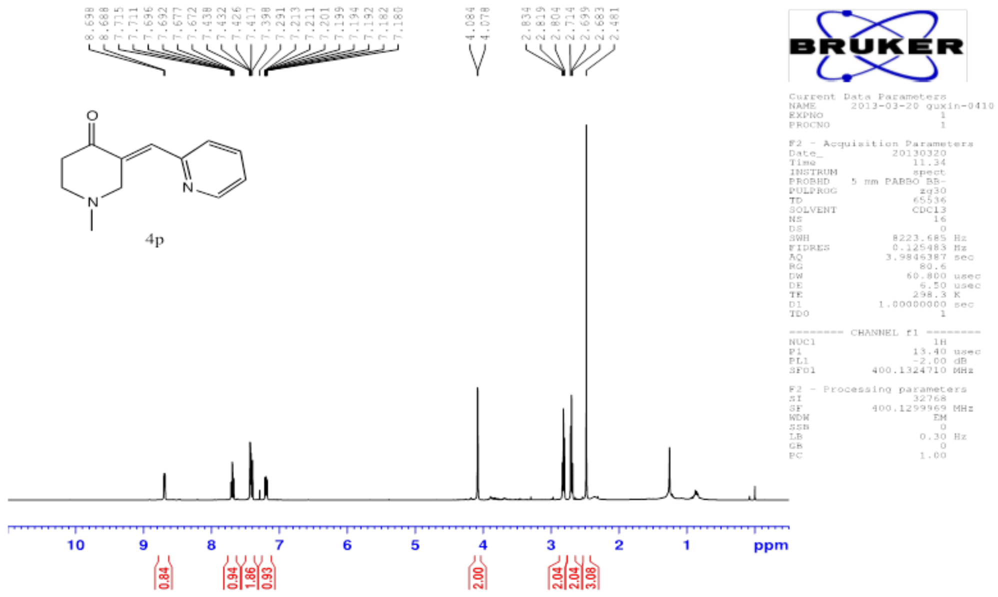

(P-1)

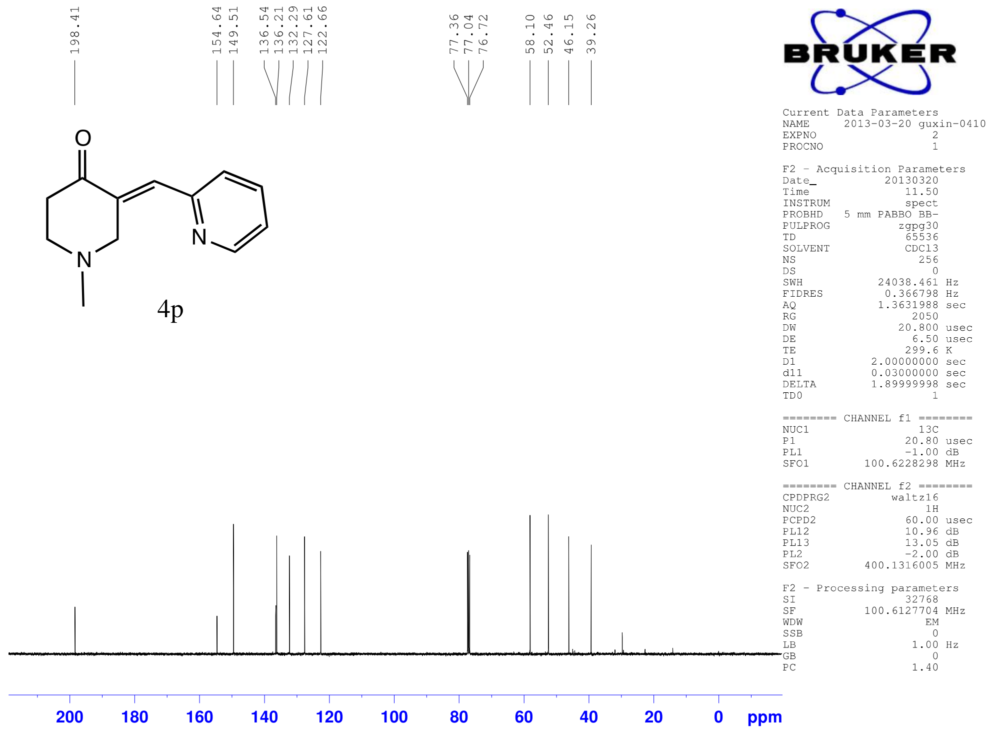

(P-2)

Figure S6. *Cont.*

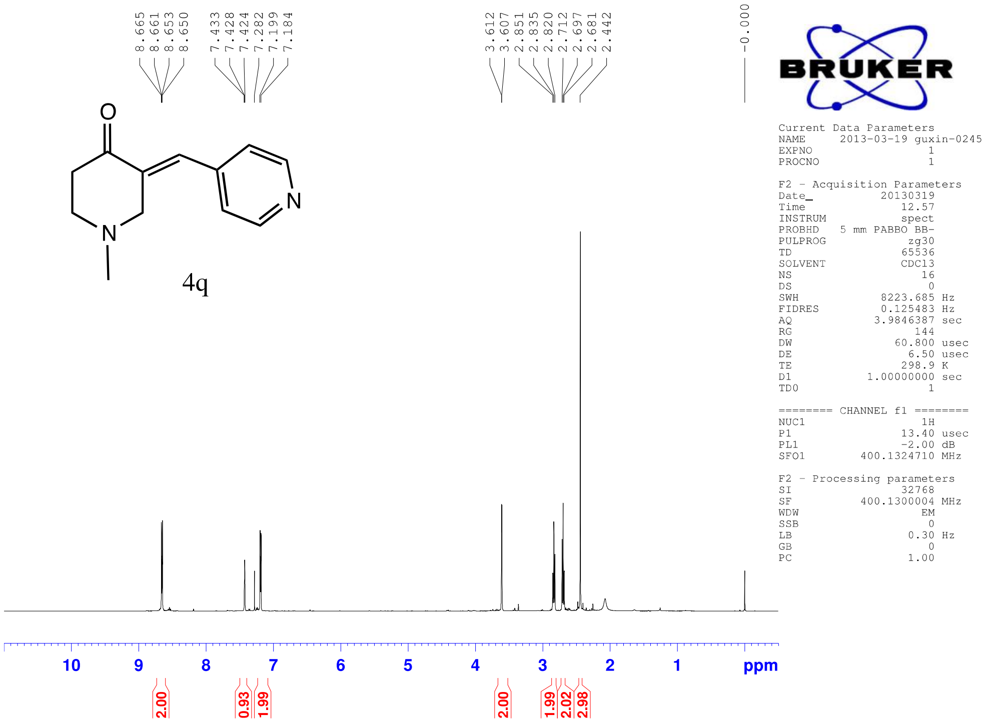

(Q-1)

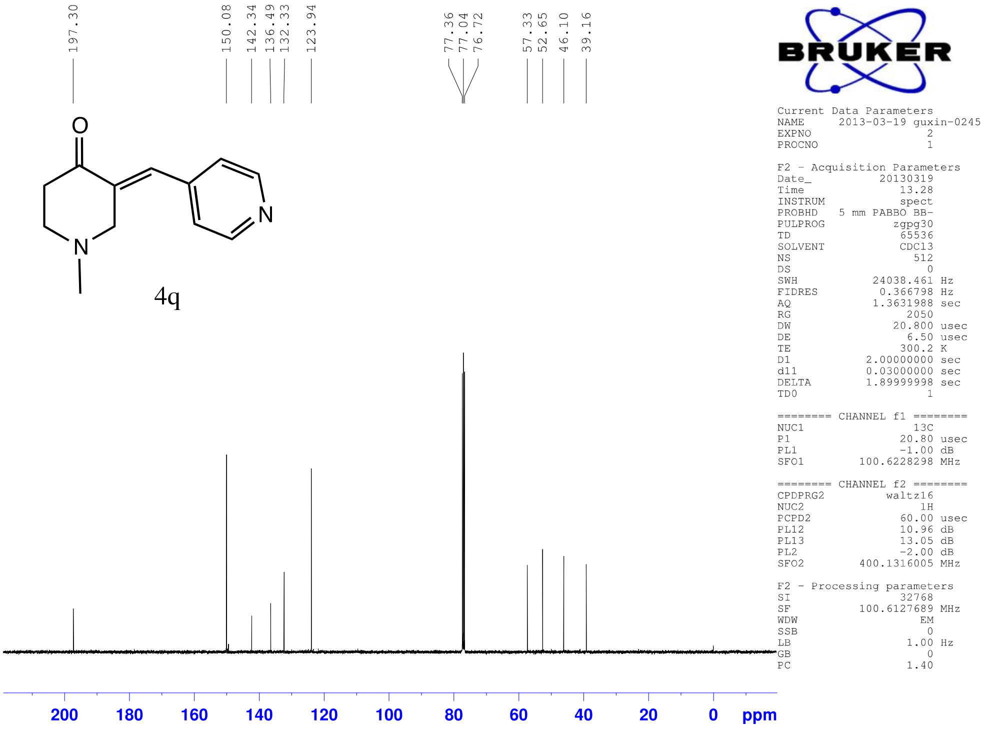

(Q-2)

Figure S7. The ^{1}H and ^{13}C-NMR spectra of **4r–t**. (**R-1**) ^{1}H-NMR spectrum of compound **4r**; (**R-2**) ^{13}C-NMR spectrum of compound **4r**; (**S-1**) ^{1}H-NMR spectrum of compound **4s**; (**S-2**) ^{13}C-NMR spectrum of compound **4s**; (**T-1**) ^{1}H-NMR spectrum of compound **4t**; (**T-2**) ^{13}C-NMR spectrum of compound **4t**.

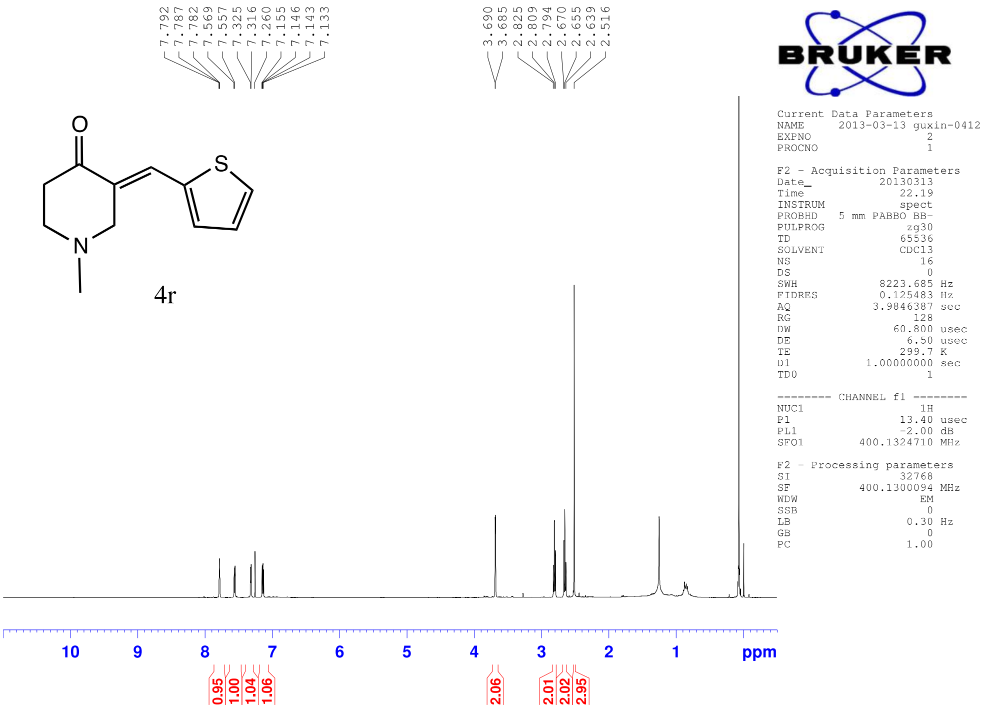

(R-1)

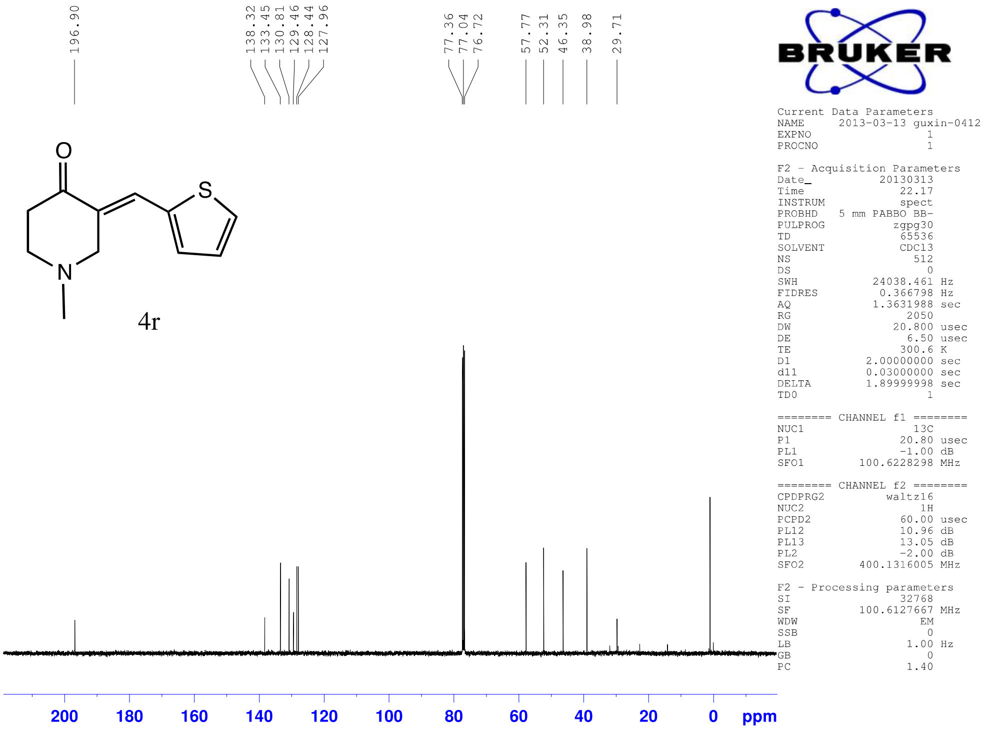

(R-2)

Figure S7. *Cont.*

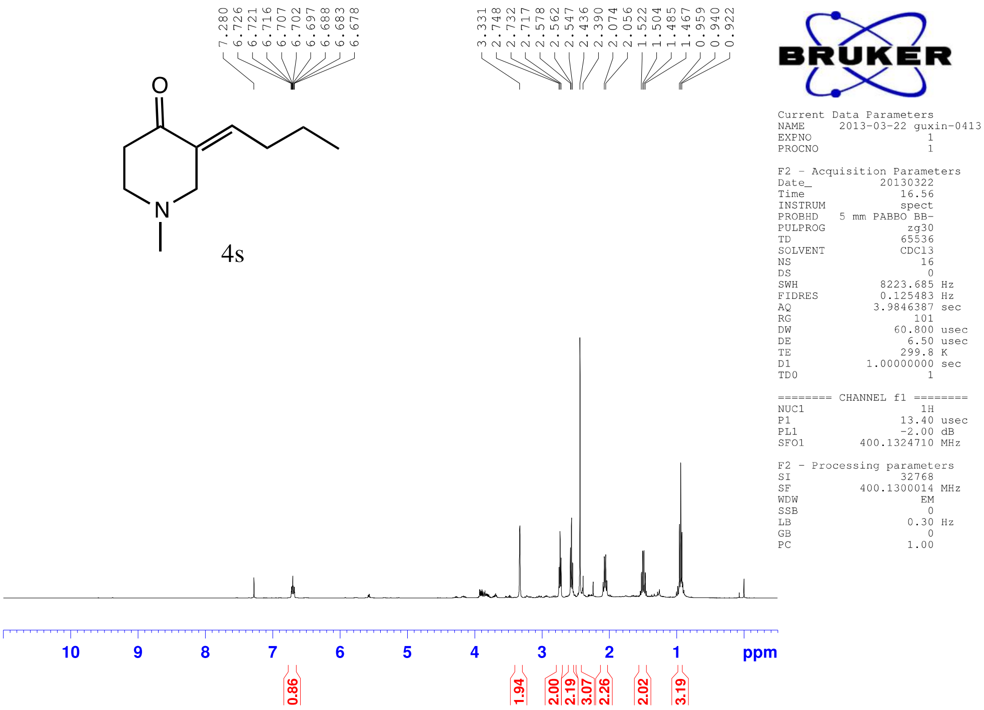

(S-1)

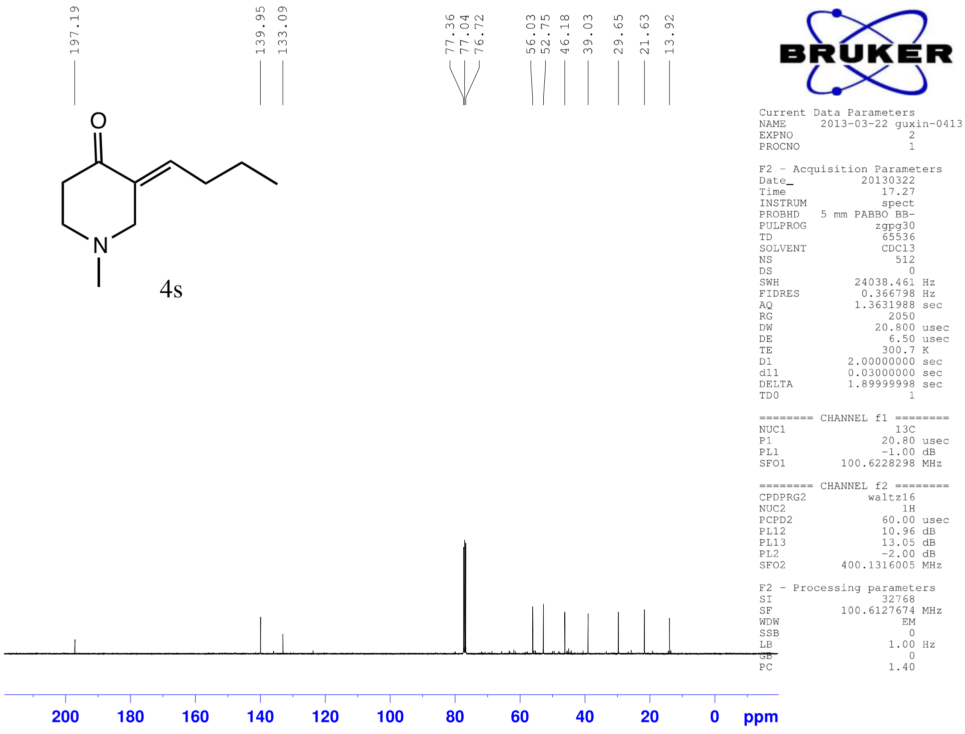

(S-2)

Figure S7. *Cont.*

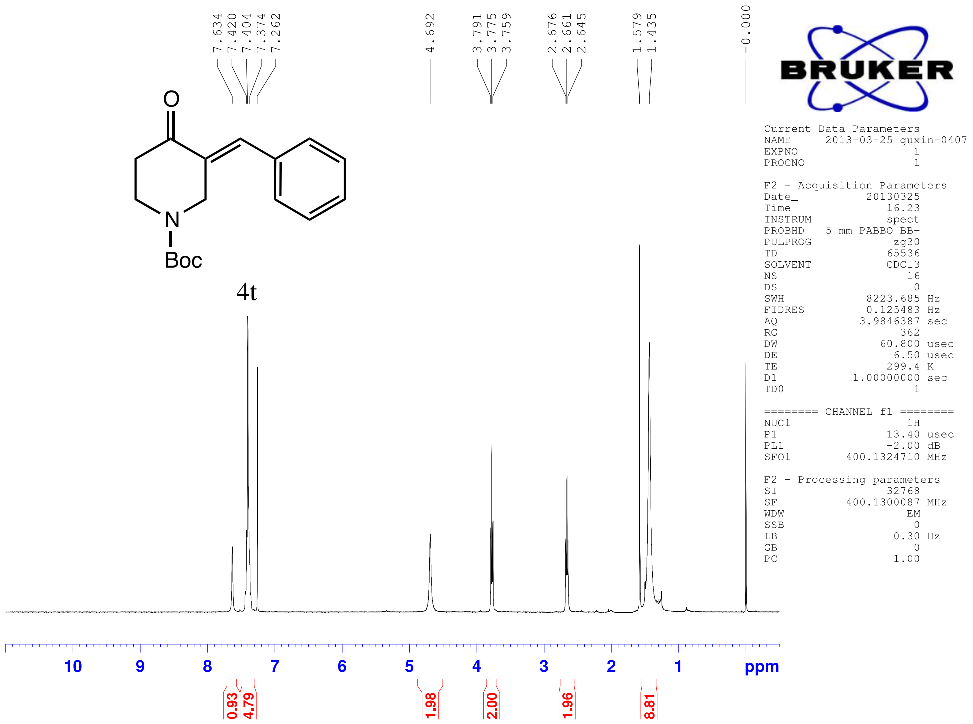

(T-1)

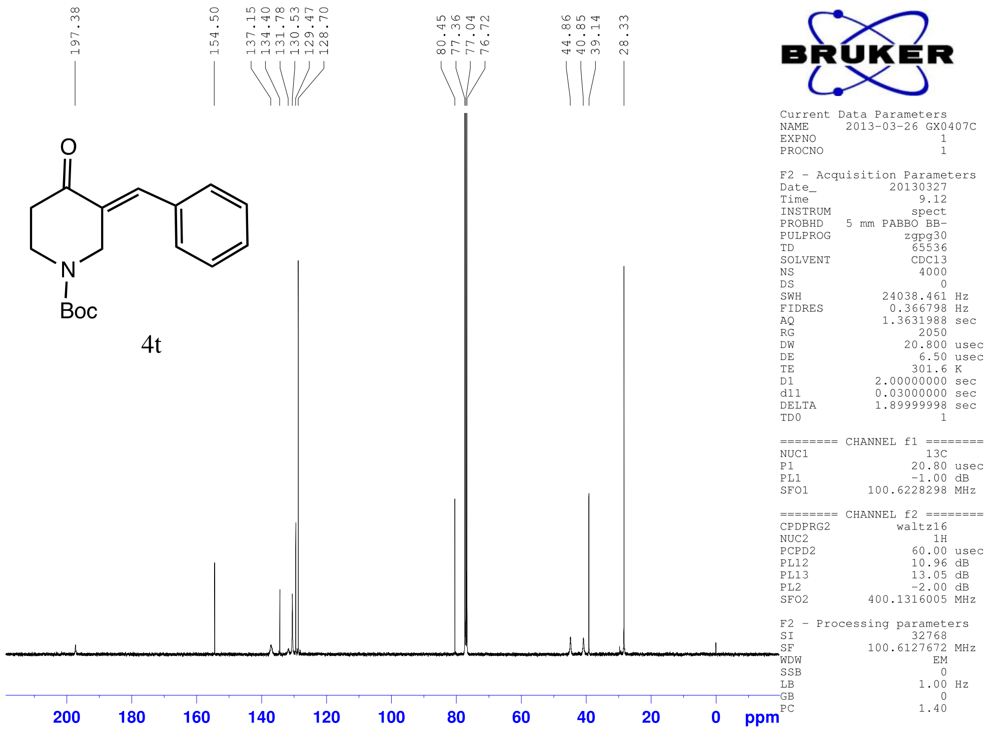

(T-2)

Figure S8. The ^{1}H and ^{13}C-NMR spectra of **4u–w**. (**U-1**) ^{1}H-NMR spectrum of compound **4u**; (**U-2**) ^{13}C-NMR spectrum of compound **4u**; (**V-1**) ^{1}H-NMR spectrum of compound **4v**; (**V-2**) ^{13}C-NMR spectrum of compound **4v**; (**W-1**) ^{1}H-NMR spectrum of compound **4w**; (**W-2**) ^{13}C-NMR spectrum of compound **4w**; (**X-1**) ^{1}H-NMR spectrum of compound **4x**; (**X-2**) ^{13}C-NMR spectrum of compound **4x**.

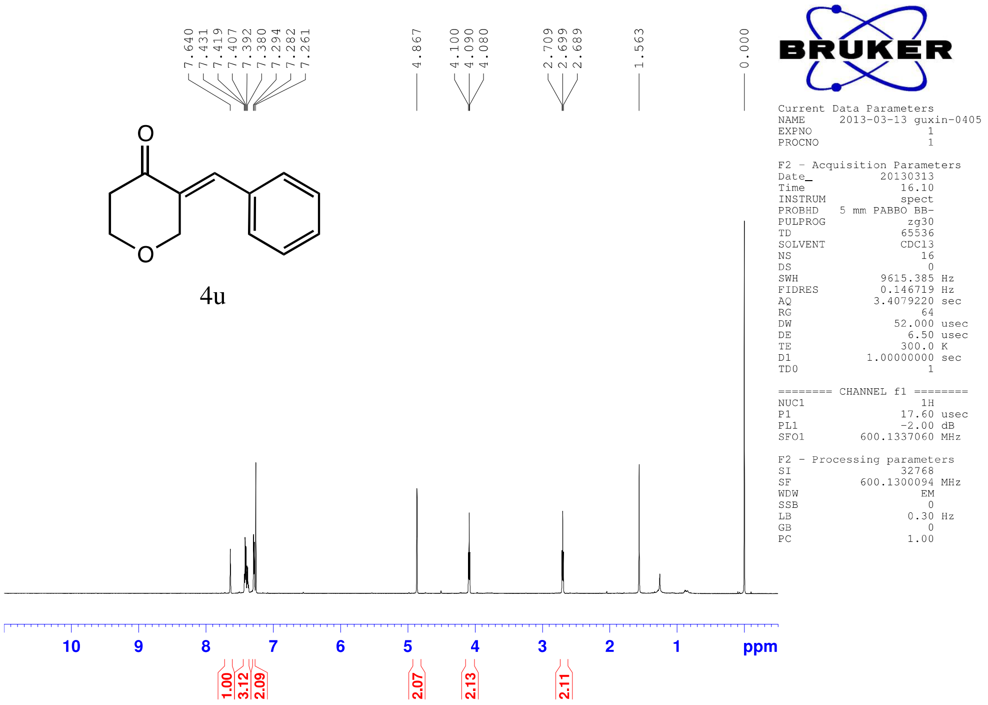

(U-1)

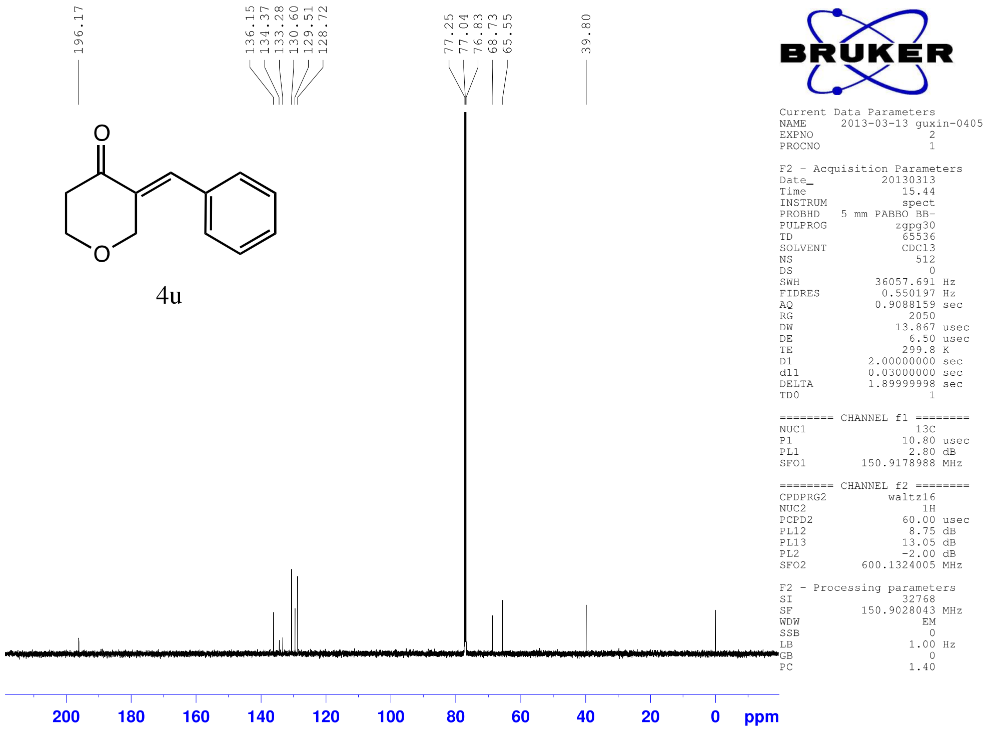

(U-2)

Figure S8. *Cont.*

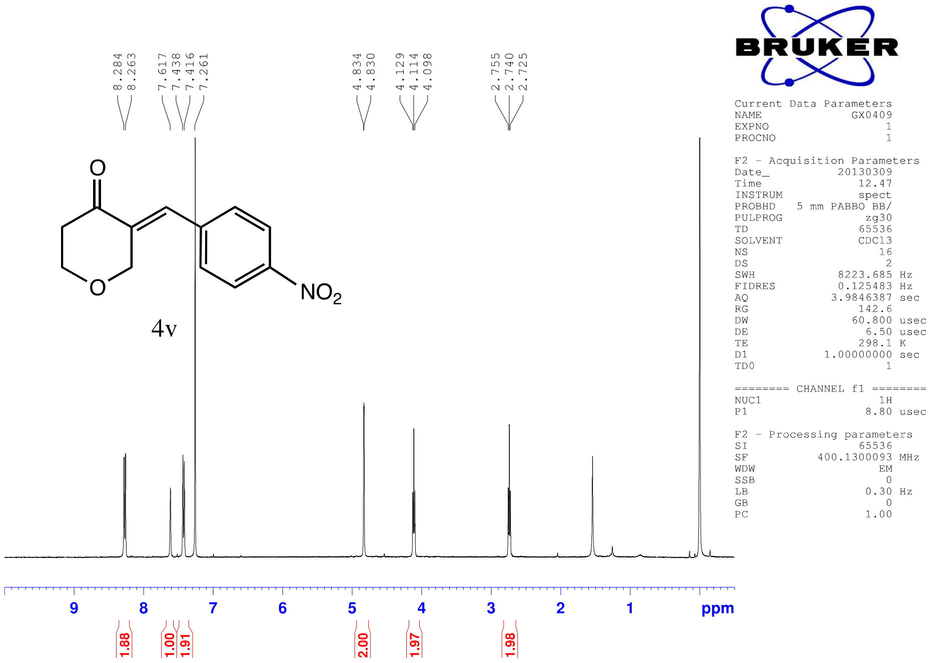

(V-1)

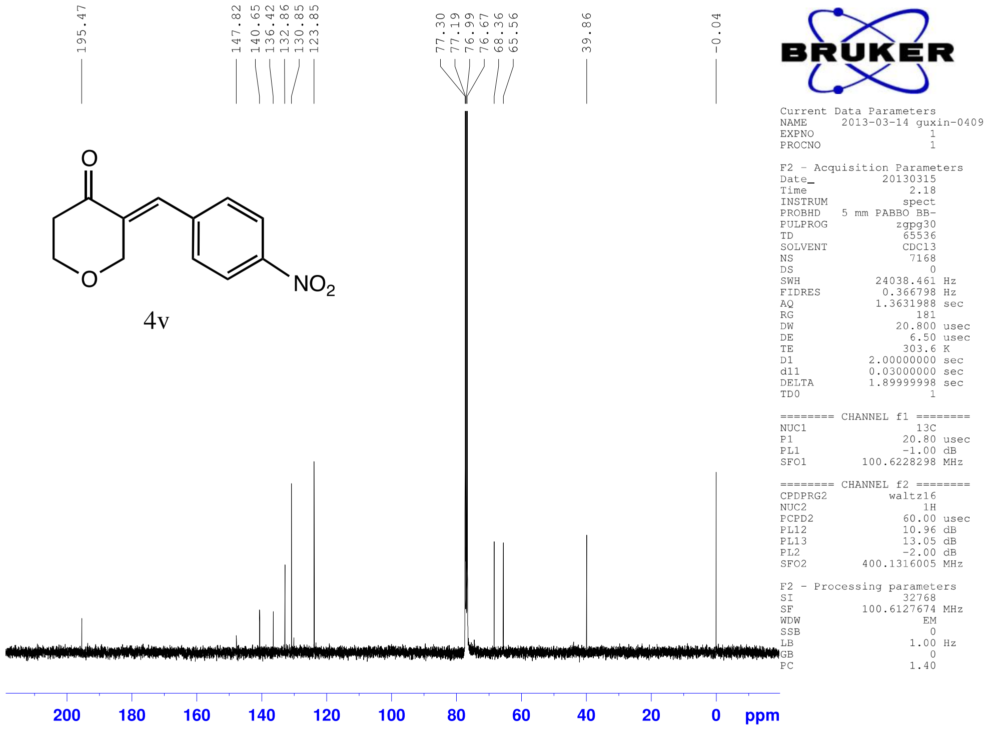

(V-2)

Figure S8. *Cont.*

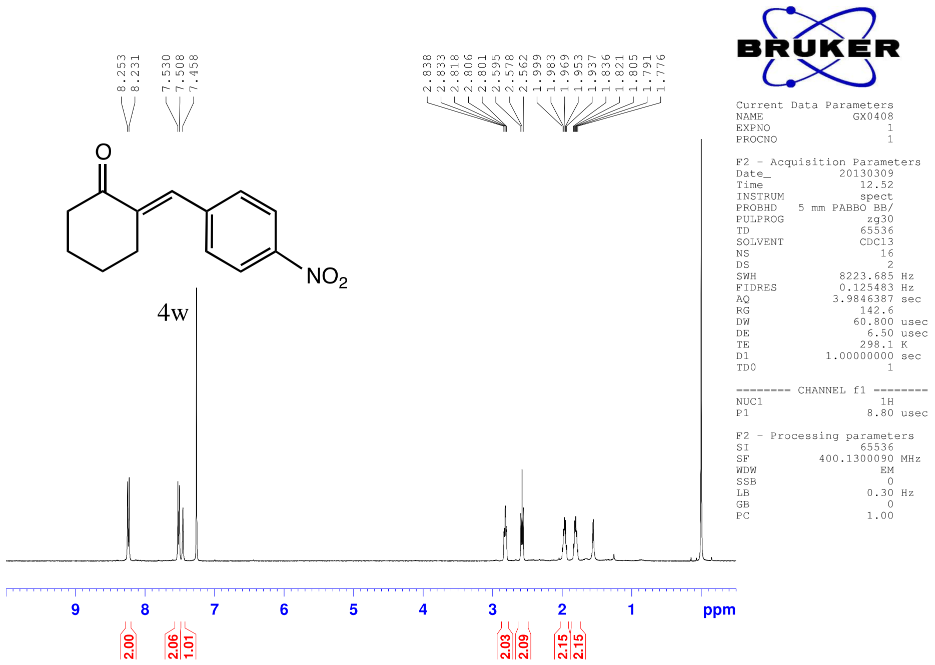

(W-1)

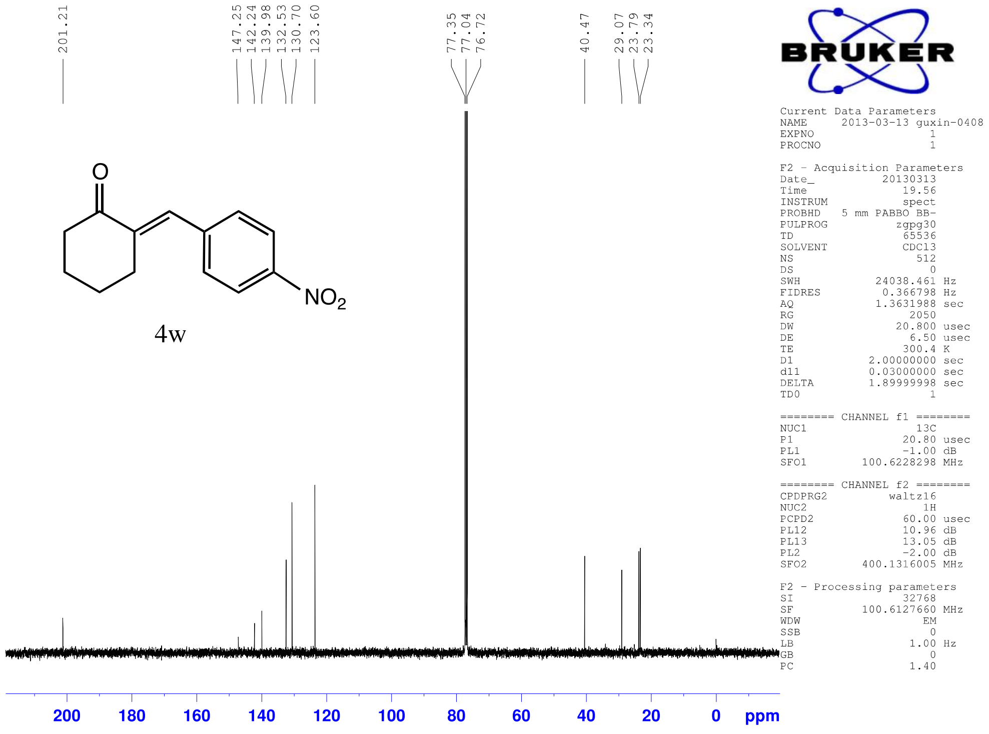

(W-2)

Figure S8. *Cont.*

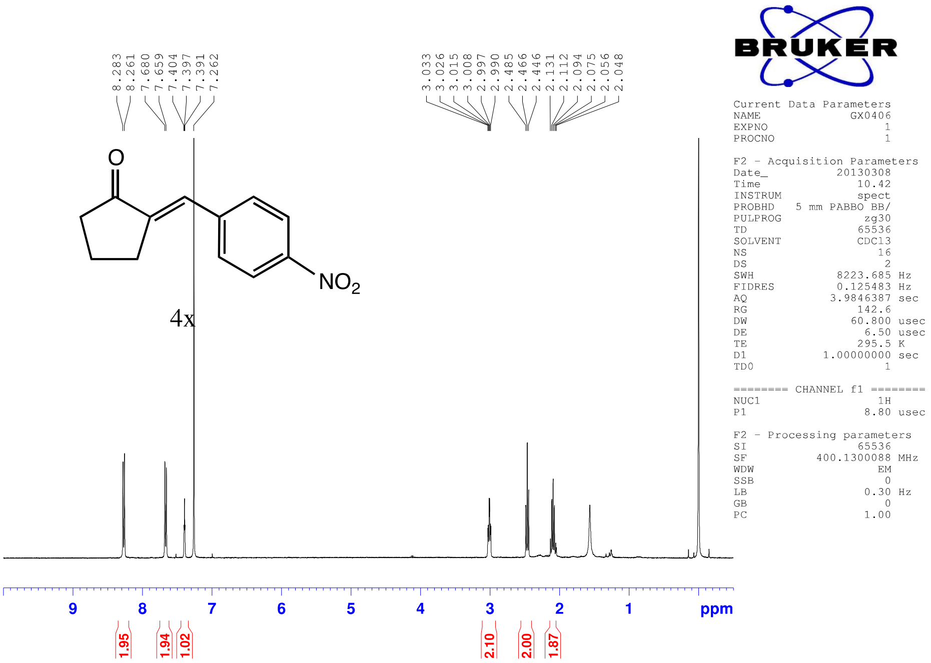

(X-1)

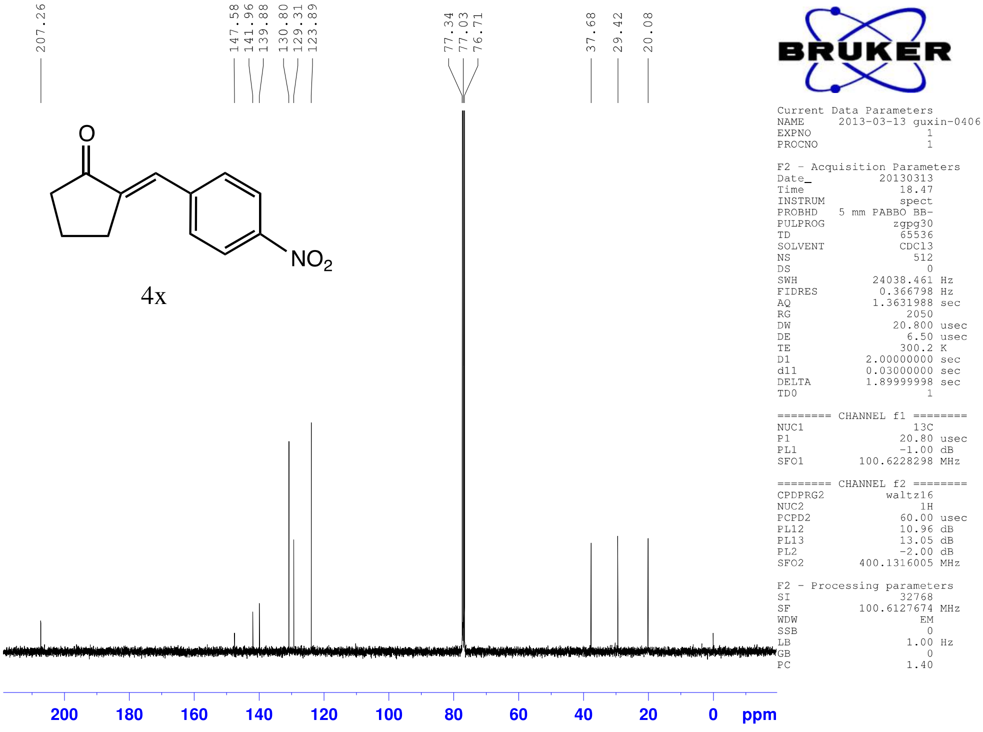

(X-2)

(E) Configuration of the Mono-Arylidene Derivatives

Compound **4a** was used as model to study the configuration. The identification of the configuration was based on NOESY spectra (Figure S9). The obvious cross-peak showed the distance between H-2 and the hydrogen in phenyl ring was less than 5Å. These results indicate the H-2 and the hydrogen in phenyl ring are on the same sides of the double bond, which therefore has the (*E*) configuration as drawn.

Figure S9. The NOESY spectrum of **4a**.

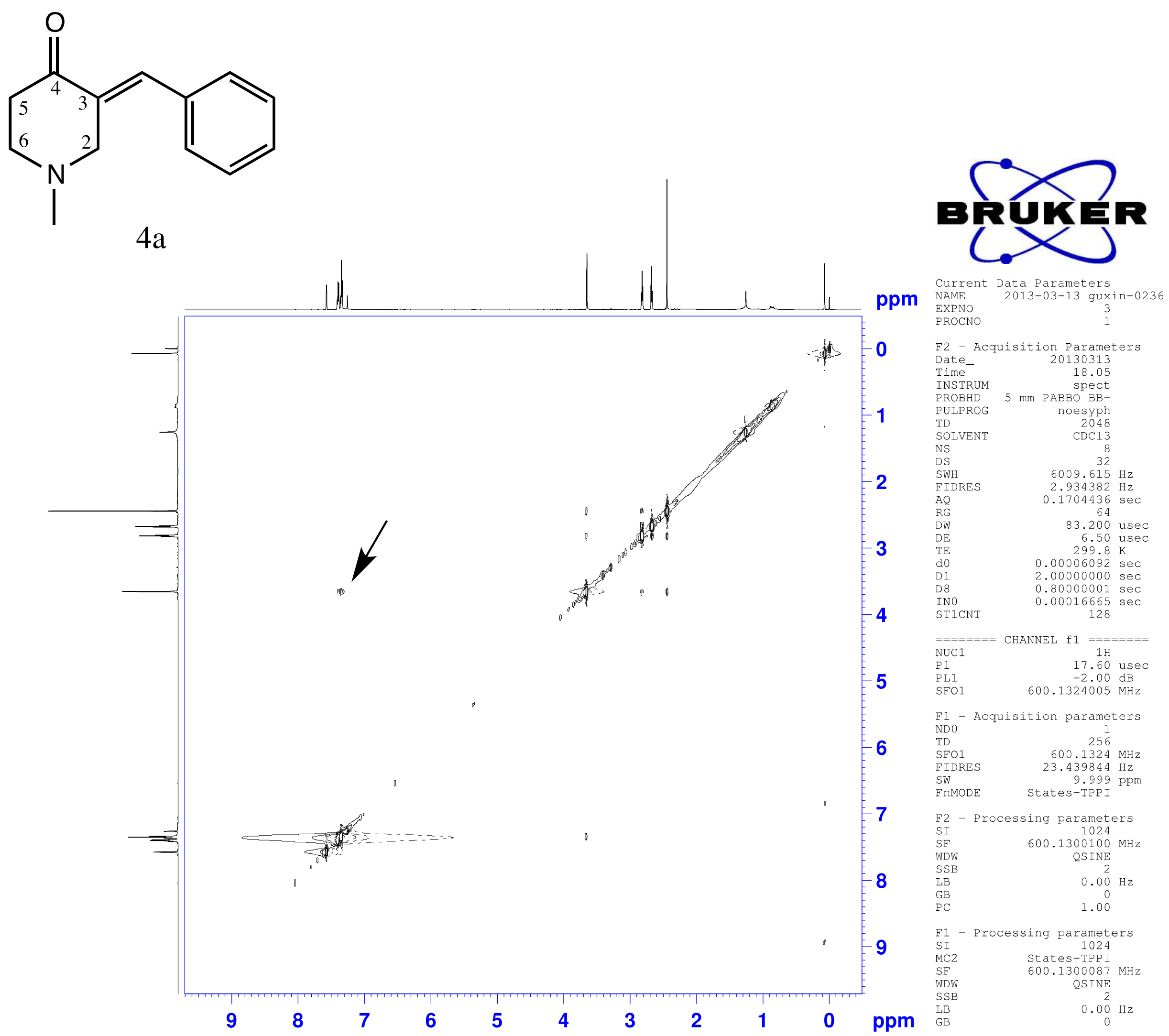

(F) Copies of NMR Spectra for Intermediate

The ^{13}C-NMR spectrum (Figure S10) of the reaction mixture, recorded after 6 h, exhibited 3 peaks at δ 209.5, 208.4 and 192.2 ppm in the range of 180–220 ppm which were assigned to carbonyl region. From HSQC spectra (Figure S10), the carbon at 64.7 ppm was a typical signal which is linked with the hydrogen at 4.62 ppm. The results of quantity ^{13}C spectra showed the integration of carbon at 209.5 ppm and the integration of carbon at 64.7 ppm were approximately equal. At the same time, the structure of intermediate c was verified by NOE (Figure S10). The obvious cross-peak between the pyrrole and the phenyl ring illustrated intermediate should be c instead of b.

Figure S10. NMR data of intermediate. (**A**) 1H-NMR spectrum of the intermediate; (**B**) The HMQC spectrum of the intermediate; (**C**) Quantity 13C-NMR spectrum of the intermediate; and (**D**) The NOESY spectrum of the intermediate.

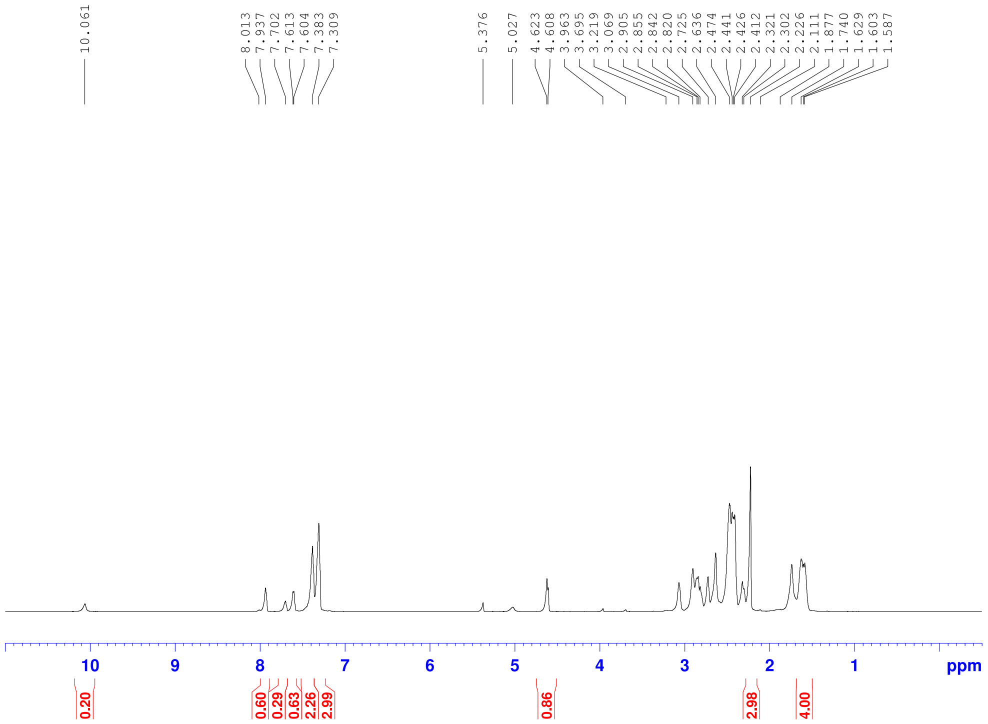

(**A**)

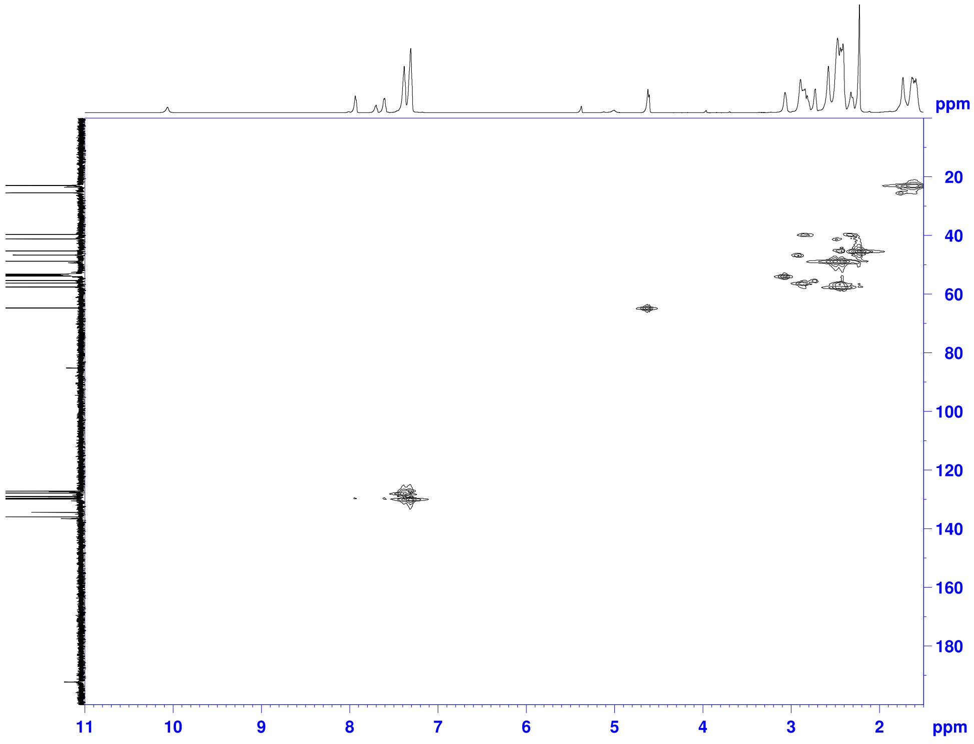

(**B**)

Figure S10. *Cont.*

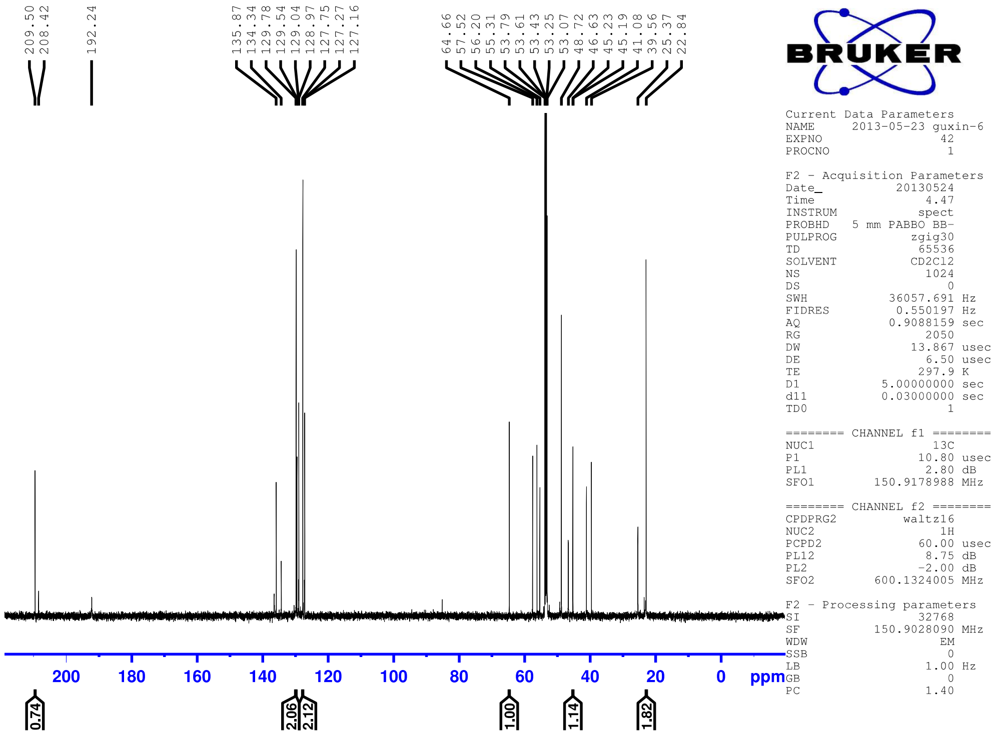

(C)

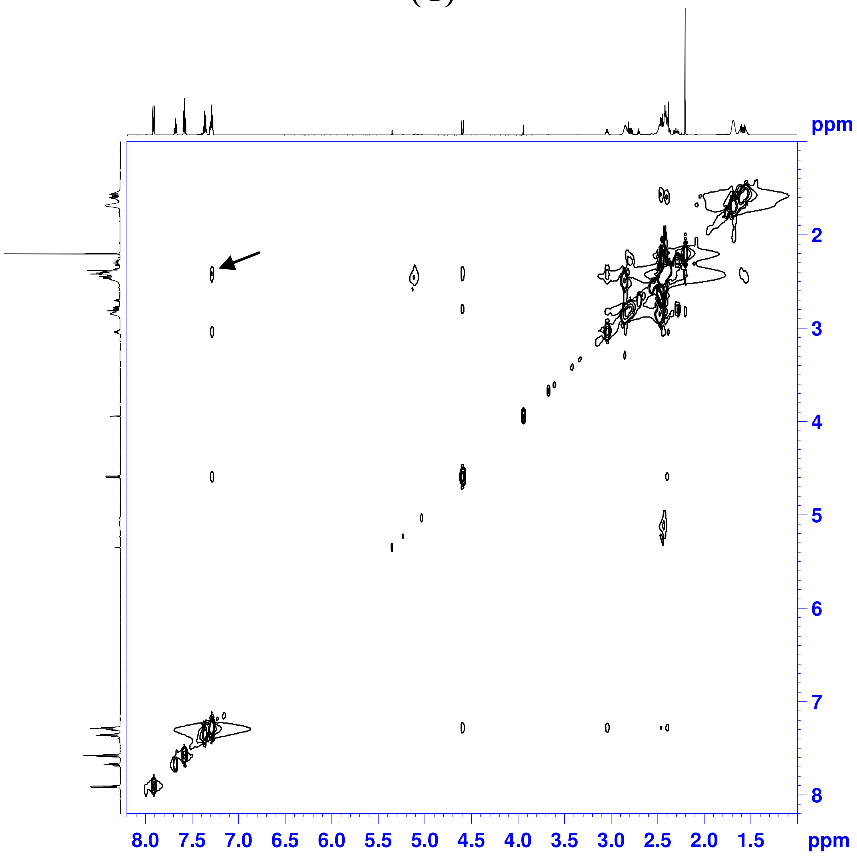

(D)
